# The Meiosis-Specific Crs1 Cyclin Is Required for Efficient S-Phase Progression and Stable Nuclear Architecture

**DOI:** 10.3390/ijms22115483

**Published:** 2021-05-22

**Authors:** Luisa F. Bustamante-Jaramillo, Celia Ramos, Cristina Martín-Castellanos

**Affiliations:** Instituto de Biología Funcional y Genómica (IBFG), Consejo Superior de Investigaciones Científicas, Universidad de Salamanca, 37007 Salamanca, Spain; luisa.fernanda.bustamante.jaramillo@gu.se (L.F.B.-J.); celiaramos125@gmail.com (C.R.)

**Keywords:** cyclins, CDK, meiosis, chromosome architecture, fission yeast

## Abstract

Cyclins and CDKs (Cyclin Dependent Kinases) are key players in the biology of eukaryotic cells, representing hubs for the orchestration of physiological conditions with cell cycle progression. Furthermore, as in the case of meiosis, cyclins and CDKs have acquired novel functions unrelated to this primal role in driving the division cycle. Meiosis is a specialized developmental program that ensures proper propagation of the genetic information to the next generation by the production of gametes with accurate chromosome content, and meiosis-specific cyclins are widespread in evolution. We have explored the diversification of CDK functions studying the meiosis-specific Crs1 cyclin in fission yeast. In addition to the reported role in DSB (Double Strand Break) formation, this cyclin is required for meiotic S-phase progression, a canonical role, and to maintain the architecture of the meiotic chromosomes. Crs1 localizes at the SPB (Spindle Pole Body) and is required to stabilize the cluster of telomeres at this location (*bouquet* configuration), as well as for normal SPB motion. In addition, Crs1 exhibits CDK(Cdc2)-dependent kinase activity in a biphasic manner during meiosis, in contrast to a single wave of protein expression, suggesting a post-translational control of its activity. Thus, Crs1 displays multiple functions, acting both in cell cycle progression and in several key meiosis-specific events.

## 1. Introduction

Cell cycle progression is governed by the modulation of CDK activity, a kinase activity formed by a catalytic subunit (CDK-Cyclin Dependent Kinase) and a regulatory subunit (cyclin). In unicellular eukaryotes such as yeasts, a unique CDK binds to several cyclins, and the cellular level of kinase activity temporally orders the different cell cycle phases, ensuring that cells first replicate their DNA before proceeding to segregate it into two identical daughter cells [1,2,3]. This general principle also applies to cell cycle progression during meiosis, the specialized cellular program that generates haploid gametes from diploid cells as an obligated reduction of the genome content prior to fertilization [4]. Given the essential and conserved function of CDK activity in cell cycle progression, it is coordinated with many aspects of the cellular physiology such as metabolism, DNA damage and cell differentiation [5,6,7,8,9,10,11,12,13].

Apart from its role in meiotic progression, CDK has been implicated in different specialized meiotic processes. In yeasts, and probably in nematodes, it is required for Double Strand Break (DSB) formation, the DNA breaks that initiate programmed recombination in meiotic prophase [14,15,16]. In addition, mouse CDK2 regulates the binding of telomeres to the nuclear membrane, promoting the so called *bouquet* configuration of the nucleus in early meiosis [17]; and this process also requires Cyclin E1 and Cyclin E2 [18,19]. Furthermore, CDK2 also controls latter events, such as crossover (CO) maturation [20,21,22], and the conserved non-conventional cyclin CNTD1 (Cyclin N-terminal domain containing protein 1) shares this function [23]. This role in CO maturation is conserved in nematodes and plants [24,25]. In the case of *A. thaliana*, CDKA;1 binds to the meiosis-specific cyclins SDS and TAM [25]. Meiosis-specific cyclins are widespread in evolution and they have also been described in *Tetrahymena*, fission yeast, and mammals [26,27,28,29]. Finally, not only cyclins, but also CDKs show peculiarities in meiosis. Mouse CDK2 shows a meiosis-boosted long spliced isoform that it is stabilized by a non-cyclin activator, and Cdk3 is a conjugation-specific CDK in *Tetrahymena* [29,30,31]. Thus, the meiotic program has exploited CDK activity for a variety of meiosis-specific events that in some cases are even regulated by meiosis-specific variants.

Fission yeast is an excellent model to study many aspects of meiosis, and CDK contribution. Six cell cycle cyclins have been identified (Cig1, Cig2, Puc1, Cdc13, Rem1 and Crs1), and their role in meiotic progression studied. As in the mitotic cycle, the main cyclin promoting DNA replication is Cig2, though redundancy compensates for the lack of this cyclin [4,32]; in contrast, Cdc13 is essential for chromosome segregations [4]. Cdc13 localizes at the Spindle Pole Body (SPB) at the end of prophase, and spreads to the spindle in meiosis I [33,34]. However, localization for the rest of the cyclins has not been reported. The only CDK present, Cdc2, appears in centromeres and the SPB during prophase; and in the SPB and the spindle during chromosomes segregations [33,34].

One of the most extensively studied topics in fission yeast meiosis is nuclear architecture and movement during meiotic prophase; and indeed, the mechanism of *bouquet* organization was first described in this organism. Upon meiosis entry, centromeres initially located at the SPB detach, and telomeres initially located at the nuclear periphery cluster to it [35,36]. This reorganization is established by the meiosis-specific proteins Bqt1 and Bqt2 that interact both with the telomeric protein Rap1 and the SPB component Sad1 to tether telomeres to the SPB [37,38]. *Bouquet* configuration is essential for the nuclear movement during prophase, known as *horsetail* movement because of its shape and extremely vigorous motion. The SPB organizes the cytoplasmic microtubules, leading to nuclear oscillations that pull the SPB-attached chromosomes between the cell poles, a process that requires proteins of the cell cortex and microtubule motors [39]. This nuclear architecture is coordinated with the DNA physiology, and the Cds1-dependent replication checkpoint and the Chk1-dependent DNA damage checkpoint coordinate *bouquet* and nuclear movements with DNA replication and recombination [34,40]. The *bouquet* plays important roles in meiosis; it is required for homologous chromosome alignment and recombination, centromere maturation, and enables the SPB to form a functional spindle [41,42,43,44]. At present, it is unknown whether CDK plays a role in *bouquet* formation in fission yeast.

Rem1 and Crs1 are the meiosis-specific cyclins of fission yeast [27,28]. Levels of these cyclins are tightly regulated, and mis-expression in vegetative cells promotes cell cycle arrest and aberrant DNA segregations. In the case of Crs1, expression in vegetative cells is downregulated by the RNA binding protein Mmi1 that promotes intron retention and polyadenylation-coupled RNA turnover [45,46]. Recently, it has been reported that the untimely expression of Crs1 during vegetative growth causes uniparental disomy, a condition linked to congenital disorders and cancer, that might originate from abnormal reductional chromosome segregations during mitotic divisions [47].

We have recently shown that Crs1 has a predominant role in DSB formation [16]. However, apart from the RNA expression pattern reported in several genome wide studies, as well as in particular studies using *crs1* as a model for regulation of meiosis-specific genes, no data about protein levels, associated kinase activity or localization have been published for this cyclin. We have addressed these issues in the present report. Crs1 protein shows an expression pattern similar to the one described for the RNA, from early S-phase to the first meiotic division. In addition, it displays Cdc2-dependent associated kinase activity. Strikingly, the kinase activity does not mirror the protein levels. There are two waves of kinase activity, one at S-phase and a higher one at meiosis I entry, suggesting a post-translational regulation of the activity during prophase and a boost of activity prior to the first meiotic division. In agreement with the first wave of activity, *crs1* deletion exacerbates the replication defect of *cig2* cyclin mutants. During meiotic prophase, Crs1 localizes throughout the nucleus in addition to the SPB, indicating additional functions. Indeed, *bouquet* formation and nuclear movements are impaired in *crs1* mutants. Thus, the meiosis-specific Crs1 cyclin seems to be a multitask cyclin with a role in several key aspects of meiosis, DNA replication, DSB formation, and nuclear architecture and dynamics.

## 2. Results

**Figure 1:**
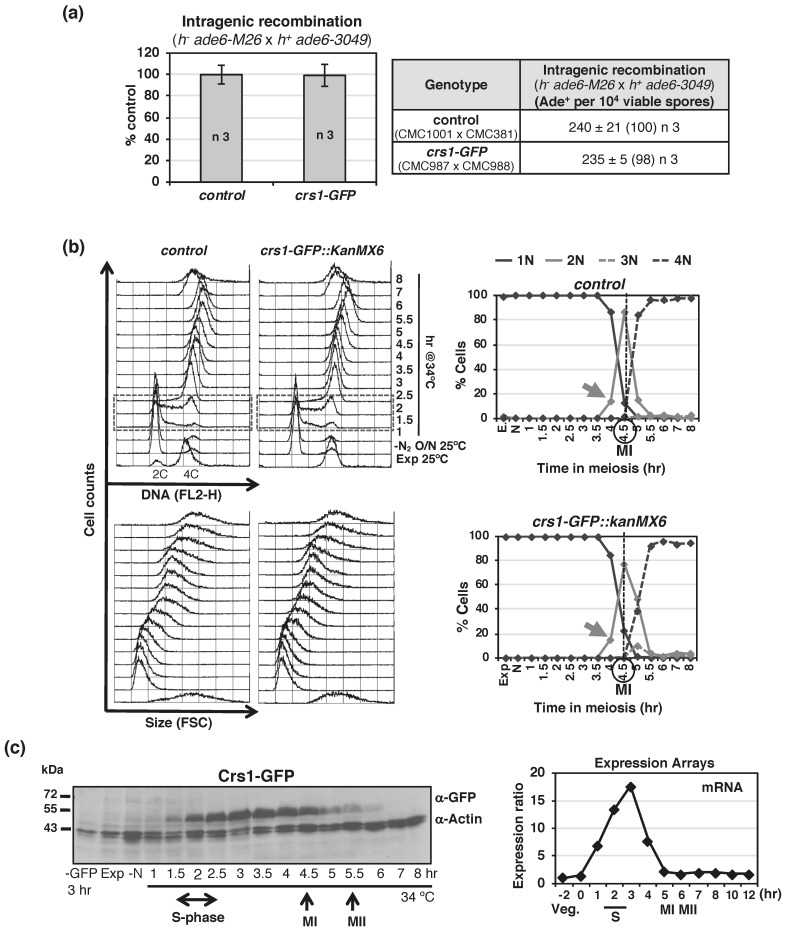
Crs1-GFP functionality and expression. (**a**) Recombination assays showing that Crs1-GFP is proficient for recombination. Crosses of *h^−^ ade6-M26* x *h^+^ ade6-3049* in MEA were performed and plated for recombinant frequency twice. Table shows gene conversion (intragenic recombination) expressed as the mean of Ade^+^ per 10^4^ viable spores +/−SEM of 3 independent crosses based on the cumulative number of spore colonies in each cross; 168–294 Ade^+^ colonies scored in each independent cross. The numbers in parentheses are percentages relative to wild-type control. Strains used in the crosses are indicated. Graph shows same data as mean of the percentage relative to the control cross +/−SEM. (**b**) Flow cytometry analysis of synchronous meiosis of diploid *pat1-114* control (CMC1074) and *pat1-114 crs1-GFP* (CMC1000) cells. DNA content (FL2-H) and Size (FSC) histograms are shown. Dashed-lined box outlines S-phase progression. On the right: Quantification of chromosome segregation by DAPI staining and nuclear counting (1 nucleus, 2 nuclei, 3 nuclei, and 4 nuclei) is shown. The arrows indicate meiosis I (MI) entry, and the vertical dashed-lines indicate the peak of MI. (**c**) Crs1-GFP protein expression during synchronous diploid *pat1-114* meiosis (CMC1000). Same blot was sequentially used with anti-GFP and anti-Actin antibodies. On the right, mRNA expression levels during synchronous diploid *pat1-114* meiosis (microarrays data from Pombase).

To characterize Crs1, we have generated a GFP-tagged version that is fully functional in terms of recombination proficiency (Figure 1a). During synchronous meiosis induced by temperature inactivation of Pat1-114 kinase [48], Crs1-GFP protein was detected continuously from S-phase to meiosis I with maximal levels during prophase (Figure 1b,c). This expression pattern corresponds well with the reported messenger profile (http://www.pombase.org/spombe/result/SPBC2G2.09c; expression viewer and pombeTV [49], last accessed date 19 February 2021).

**Figure 2:**
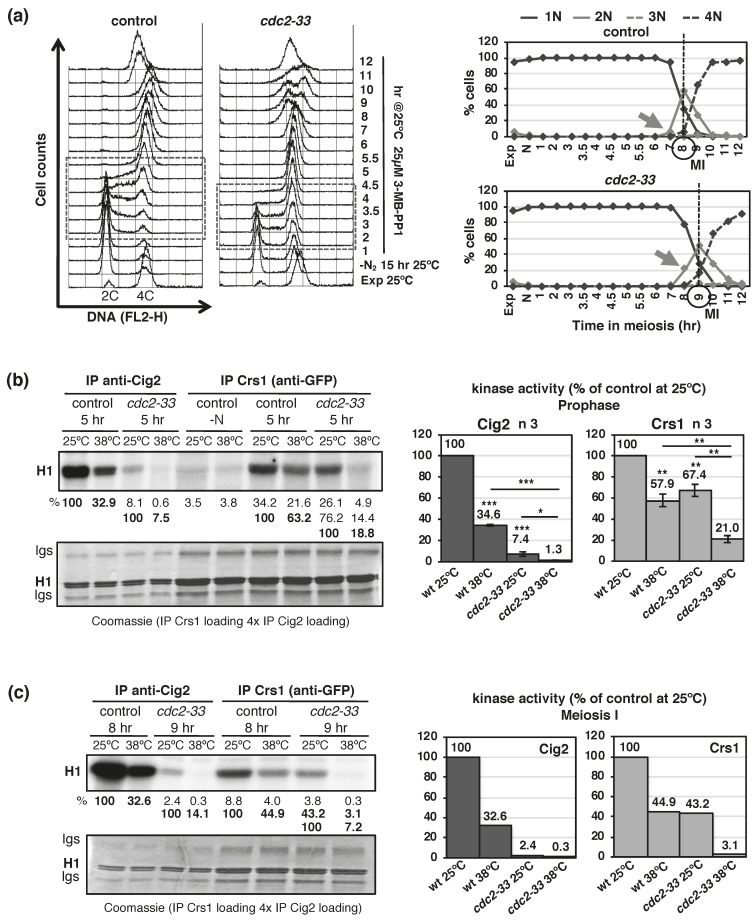
Crs1-GFP shows Cdc2-dependent kinase activity. (**a**) Flow cytometry analysis of synchronous diploid *pat1-as1(L95G) crs1-GFP* control (CMC1098) and *cdc2-33* (CMC1109) meiosis at 25 °C, 3-MB-PP1 ATP-analog added at the beginning of meiotic induction. DNA content (FL2-H) histograms are shown. Dashed-lined box outlines S-phase progression. On the right: Quantification of chromosome segregation by DAPI staining and nuclear counting (1 nucleus, 2 nuclei, 3 nuclei, and 4 nuclei) is shown. The arrows indicate meiosis I (MI) entry, and the vertical dashed-lines indicate the peak of MI. (**b**) In vitro kinase assays (histone H1 substrate) of Cig2 and Crs1-GFP immunoprecipitates (IP) at meiotic prophase (5 h after meiotic induction) of the synchronous diploid *pat1-as1(L95G) crs1-GFP* kinetics shown in (**a**). IPs were split in two just before the assay, and tested for kinase activity at 25 °C and 38 °C (permissive and restrictive temperature for *cdc2-33* mutation). In the case of Crs1-GFP, cells at the beginning of the kinetics (nitrogen depleted, -N) were also collected as a control. The top panel shows phosphorimager scanning of the assays ran in an acrylamide gel, and percentages of activity. The bottom panel shows Coomassie staining of the same gel. Notice the 4-fold higher loading of the Crs1-GFP assays. Graphs on the right represent Cig2 and Crs1-associated prophasic-kinase activity as the mean of the percentage of activity relative to the *cdc2^+^* control at 25 °C +/−SEM of 3 independent assays. The assays correspond to 2 replicates from 2 independent aliquots of frozen cells assayed in different days, and 1 replicate from a different time course. *p* value * < 0.05, ** ≤0.01, *** ≤0.001 (Student’s *t*-test, unpaired, two tails). (**c**) In vitro kinase assays (histone H1 substrate) of Cig2 and Crs1-GFP immunoprecipitates (IP) at meiosis I (8–9 h after meiotic induction). IPs were split in two just before the assay, and tested for kinase activity at 25 °C and 38 °C (permissive and restrictive temperature for *cdc2-33* mutation). Similar data representation as in (**b**). Graphs on the right represent Cig2 and Crs1-associated kinase activity as the percentage of the activity observed in the *cdc2^+^* control at 25 °C.

Next, we addressed its putative kinase activity in prophase, when maximal expression and DSB formation occurs. Kinase activity was measured in vitro using histone H1 as a substrate, and a *cdc2-33* temperature sensitive allele that allowed us to compare kinase activity at permissive and restrictive temperature to address Cdc2-dependency [50,51,52]. Synchronous meiosis of control and *cdc2-33* mutant were induced by chemical inhibition (3-MB-PP1) of the Pat1-as1 (L95G) kinase at 25 °C (Figure 2a) [53]. Extracts from cells collected at the beginning of meiosis (-N) and at 5 h after meiotic induction (prophase) were immunoprecipitated with antibodies anti-GFP (Crs1-GFP) and anti-Cig2 as a control, split in two, and assayed for kinase activity at 25 °C (*cdc2-33* permissive temperature) and 38 °C (*cdc2-33* restrictive temperature) (Figure 2b). As previously reported [54,55], Cig2 cyclin showed associated kinase activity that was temperature sensitive (kinase activity in *cdc2^+^* control cells at 38 °C was 34.6% +/−0.9 SEM of that observed at 25 °C, *p* value 2.1 × 10^−7^ ), and Cdc2-dependent (kinase activity in *cdc2-33* mutant cells at 25 °C was 7.4% +/−2.0 SEM of that observed in *cdc2^+^* control cells, *p* value 1.4 × 10^−6^; and it was drastically reduced to 1.3% +/−0.4 SEM in *cdc2-33* mutant cells at 38 °C, *p* value 4.2 × 10^−6^). Interestingly, we did detect kinase activity associated to Crs1, though less activity than the one detected in Cig2 immunoprecipitates (6.87% +/−0.86 SEM, *p* value 0.0043). Indeed, in order to have alike signals to compare and quantify reliably, Crs1 assays were loaded 4-folds compared to Cig2 assays. As for Cig2, Crs1 showed associated kinase activity that was temperature sensitive and Cdc2-dependent (Figure 2b). Kinase activity in *cdc2^+^* control cells at 38 °C was 57.9% +/−6.0 SEM of that observed at 25 °C, *p* value 0.0021; kinase activity in *cdc2-33* mutant cells at 25 °C was 67.4% +/−5.8 SEM of that observed in *cdc2^+^* control cells at the same temperature, *p* value 0.0048; and it was strongly reduced to 21.0% +/−3.3 SEM in *cdc2-33* mutant cells at 38 °C, *p* value 0.0056. The temperature sensitivity of the kinase activity associated to these cyclins is not a consequence of the high temperature in the assay (38 °C) since cyclin Cdc13-associated kinase activity is not temperature sensitive (Figure S1).

**Figure 3:**
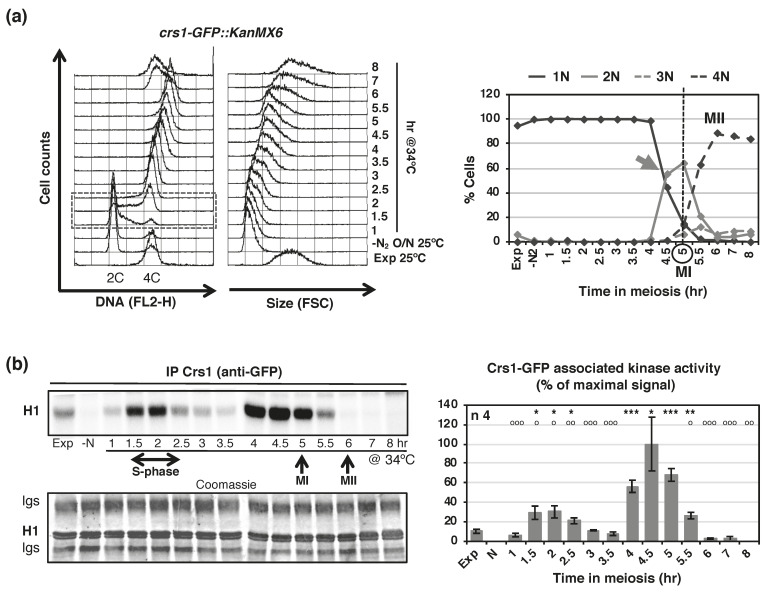
Crs1-GFP shows periodic associated kinase activity. (**a**) Flow cytometry analysis of synchronous meiosis of diploid *pat1-114 crs1-GFP* cells (CMC1000). DNA content (FL2-H) and Size (FSC) histograms are shown. Dashed-lined box outlines S-phase progression. On the right: Quantification of chromosome segregation by DAPI staining and nuclear counting (1 nucleus, 2 nuclei, 3 nuclei, and 4 nuclei) is shown. The arrow indicates meiosis I (MI) entry, and the vertical dashed-line indicates the peak of MI. (**b**) Crs1-GFP associated kinase activity during synchronous diploid *pat1-114* meiosis shown in (**a**). The top panel shows phosphorimager scanning of the assays ran in acrylamide gels. The bottom panel shows Coomassie staining of the same gels. Graph on the right represents kinase activity as the mean of the percentage relative to the maximal signal +/−SEM of 4 independent experiments. Time points during the meiotic time course (from 1 h to 8 hr) were compared to 1 h (*) and 4 h signal (^o^). * comparison shows the statistical definition of the two peaks of activity at S-phase and meiosis I entry. ^o^ comparison shows the statistical difference between S-phase and meiosis I peaks. *p* value * and ^o^ < 0.05, ** and ^oo^ ≤ 0.01, *** and ^ooo^ ≤ 0.001 (Student´s *t*-test, unpaired, two tails). Only statistical differences are indicated.

Next, we decided to study Crs1-associated kinase activity along a complete synchronous meiosis. Surprisingly, we detected two waves of Crs1-associated kinase activity, one at S-phase and a higher one before meiosis I entry, with good statistical definition of both peaks and statistical difference between peaks (Figure 3). Thus, we reevaluated Cdc2-dependency at one of these waves, meiosis I entry. As shown in Figure 2c, Cdc2-dependency was confirmed; Crs1-associated kinase activity in *cdc2-33* mutant cells at 25 °C dropped to 43% of the control at the same temperature, and to 3% in the mutant at 38 °C. The kinetics of Crs1-asociated kinase activity was very different from the reported expression of the RNA and the protein (http://www.pombase.org/spombe/result/SPBC2G2.09c, expression viewer and pombeTV [49], last accessed date 19 February 2021; and Figure 1), indicating that Crs1-associated kinase activity may have a strong post-translational regulation.

**Figure 4:**
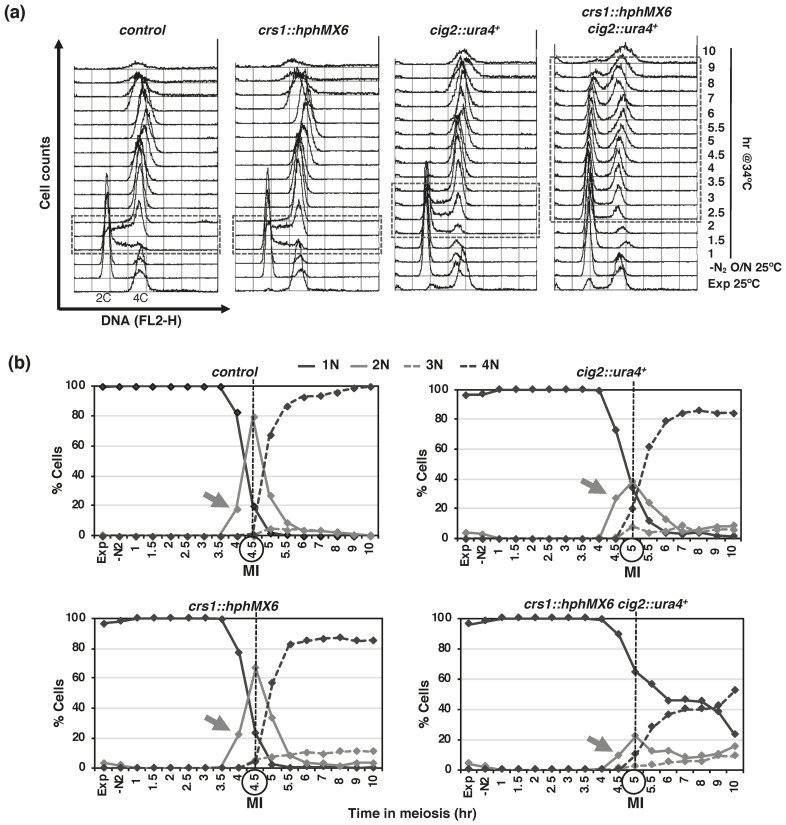
Meiosis progression of *cig2* and *crs1* mutants. (**a**) Flow cytometry analysis of synchronous diploid *pat1-114* meiosis of control (CMC1074), *crs1* (CMC1059), *cig2* (CMC1022), and double *crs1 cig2* (CMC1131) deletion mutants. DNA content (FL2-H) histograms are shown. Dashed-lined box outlines S-phase progression. (**b**) Quantification of chromosome segregation by DAPI staining and nuclear counting (1 nucleus, 2 nuclei, 3 nuclei, and 4 nuclei) is shown. The arrows indicate meiosis I (MI) entry, and the vertical dashed-lines indicate the peak of MI. Related to Figure S2.

Given the observed kinase activity during S-phase, we decided to explore the role of Crs1 in S-phase progression. *crs1* deleted cells do not have problems in S-phase entry or progression [16] (Figure 4 and Figure S2). However, we decided to use a *cig2* cyclin mutant defective in premeiotic S-phase to hamper (weaken) the process [4,16,32,56]. The defects of *cig2* deletion mutants were enhanced in the absence of *crs1*, and S-phase entry and progression were severely delayed (Figure 4a and Figure S2a). Double *crs1 cig2* deletion mutants entered S-phase later (half an hour) than single *cig2* deletion mutants, and cells in G1 were observed up to 9–10 h after meiotic induction, meanwhile *cig2* mutant cells finished replication much earlier at 3.5 h after meiotic induction. The delay in S-phase entry correlated with a delay in meiosis I (Figure 4b and Figure S2b), and indeed, *crs1 cig2* cells proceeded through meiotic divisions quite asynchronously. Thus, cyclin Crs1 contributes to S-phase progression in the absence of *Cig2*.

**Figure 5:**
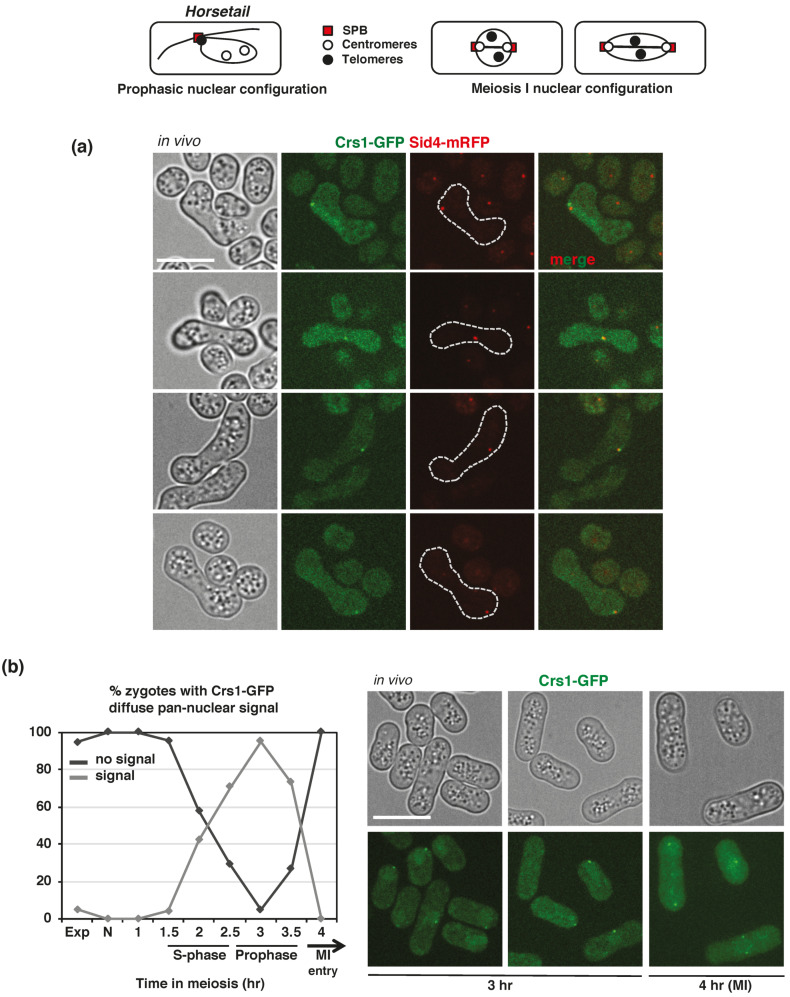
In vivo Crs1-GFP and Sid4-mRFP localization. Top panels: Schematic drawing of SPB, centromeres, and telomeres position during prophase and meiosis I is shown. (**a**) In vivo Crs1-GFP and Sid4-mRFP (SPB component) co-localization in prophase in *h^90^* zygotes (CMC1076). Single plane bright-field images on the left help to localize the nucleus, which appears as a smooth area more or less stretched depending on the nuclear movement in a rugged context. Notice the Crs1-GFP pan-nuclear diffuse signal in the upper zygote. Crs1-GFP focus and pan-nuclear signal were not always visible in the same focal plane. Crs1-GFP images correspond to single planes, and Sid4-mRFP images correspond to maximal projections of Z sections. In some zygotes, the co-localization was not perfect due to the quite different exposure time to detect the signals (2.5 s for Crs1-GFP and 0.2 s for Sid4-mRFP) and the vigorous nuclear movements at this phase of meiosis. (**b**) Crs1-GFP pan-nuclear signal during synchronous diploid *pat1-114* meiosis (CMC1000). Graph represents the percentage (mean of 2 independent experiments) of zygotes with and without Crs1-GFP diffuse pan-nuclear signal during the time course. On the right: Photographs of cells in prophase (3 h after meiotic induction) and meiosis I entry (4 h) are shown. Single plane bright-field images on top help to position the nucleus, which appears as a smooth area often distally located depending on the nuclear movement in a rugged context. Apart from the Crs1-GFP pan-nuclear signal, notice that the Crs1-GFP focus (SPB) is also visible in most of the cells. Pan-nuclear and focus Crs1-GFP signal were not always visible in the same plane. Images correspond to single planes. At least a total of 40 cells were analyzed at each time point, increasing the number of cells to 174–274 from 1.5 to 3.5 h. Scale bar corresponds to 5 µm in all the images in the figure.

**Figure 6:**
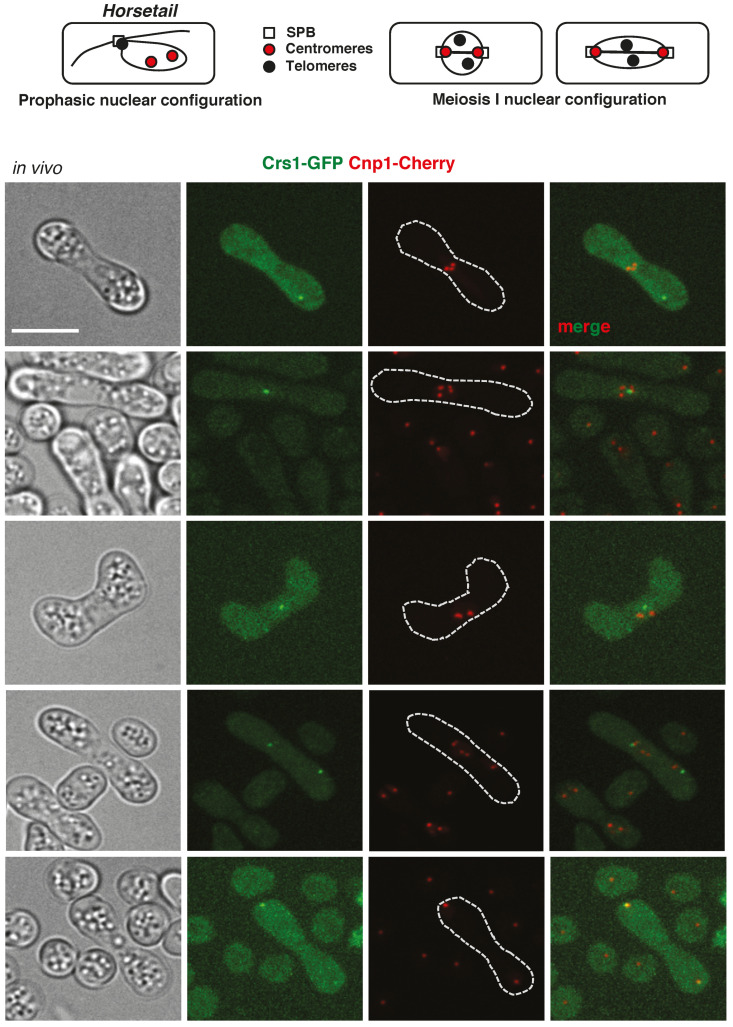
In vivo Crs1-GFP and Cnp1-Cherry localization. Top panels: Schematic drawing of SPB, centromeres, and telomeres position during prophase and meiosis I is shown. Bottom panels: In vivo Crs1-GFP and Cnp1-Cherry (centromere component) distribution in prophase and meiosis I in *h^90^* zygotes (CMC1073). Single plane bright-field images on the left help to localize the nucleus, which appears as a smooth area more or less stretched depending on the nuclear movement in a rugged context. Notice Crs1-GFP pan-nuclear diffuse signal in the upper zygote. Crs1-GFP focus and pan-nuclear signal were not always visible in the same focal plane. Notice co-localization of Crs1-GFP and Cnp1-Cherry only at the cell poles in anaphase I (when centromeres of the segregating chromosomes reach the SPB). Crs1-GFP images correspond to single planes, and Cnp1-Cherry images correspond to maximal projections of Z sections. Scale bar corresponds to 5 µm.

Finally, we studied Crs1-GFP localization. In *h^90^* zygotic meiosis, Crs1-GFP appeared as a discrete focus in what seemed to be the nuclear periphery (Figure 5). This was particular clear in zygotes in *horsetail* (prophase) when the nucleus is stretched due to vigorous movements that position it laterally in the cell; in this situation, the focus was observed facing the cortex of the cell, in what is considered the leading edge of the movement. In some nuclei, a diffuse pan-nuclear signal was also detected. In order to test whether the observed focus was the SPB, as suggested by its position, we analyzed the localization of Crs1-GFP and Sid4-mRFP (SPB component) [57], and Crs1-GFP and Cnp1-Cherry (centromere component) [58]. Nuclear architecture upon meiosis entry is very dynamic, and centromeres and telomeres exchange their mitotic nuclear position in order to build the conserved *bouquet* configuration, where centromeres initially located at the SPB are released, and telomeres initially located at the nuclear periphery are attached to it [35,36,37,38]. At the end of prophase, prior to chromosome segregations, *bouquet* structure is disorganized, and centromeres attached to the spindle reach the duplicated SPB at the cell ends in meiosis I [59] (see Figure 5 for schematic representation). Crs1-GFP focus co-localized with Sid4-mRFP in *horsetail* zygotes (Figure 5a and Figure S6). However, meanwhile Crs1-GFP focus did not co-localize with Cnp1-Cherry at this stage of meiosis (Figure 6), and indeed, in cells with elongated nuclei Crs1-GFP focus was distantly placed in front of the Cnp1-Cherry foci, Crs1-GFP and Cnp1-Cherry co-localized at the end of meiosis I when centromeres reach the SPBs (Figure 6). These data indicate that the localization of the Crs1-GFP cyclin during meiosis is similar to that of a SPB component.

To better describe the pan-nuclear signal, we decided to address Crs1-GFP localization in *pat1-114* synchronous meiosis (Figure 5b). Cells started to accumulate Crs1-GFP signal at 2 h after meiotic induction when progressing through S-phase, with a 40% of the population showing the diffuse pan-nuclear signal; at 3 h after meiotic induction (prophase) the percentage rose to 95% of the cells, and the signal disappeared when cells were entering meiosis I (4 h after meiotic induction). Meanwhile the pan-nuclear signal appeared and disappeared during the kinetics, the SPB-focus was present from the beginning of the kinetics, and duplicated as cells entered meiosis I (Figure 5b). The timing of the pan-nuclear Crs1-GFP signal is compatible with a role in S-phase progression and DSB formation.

**Figure 7:**
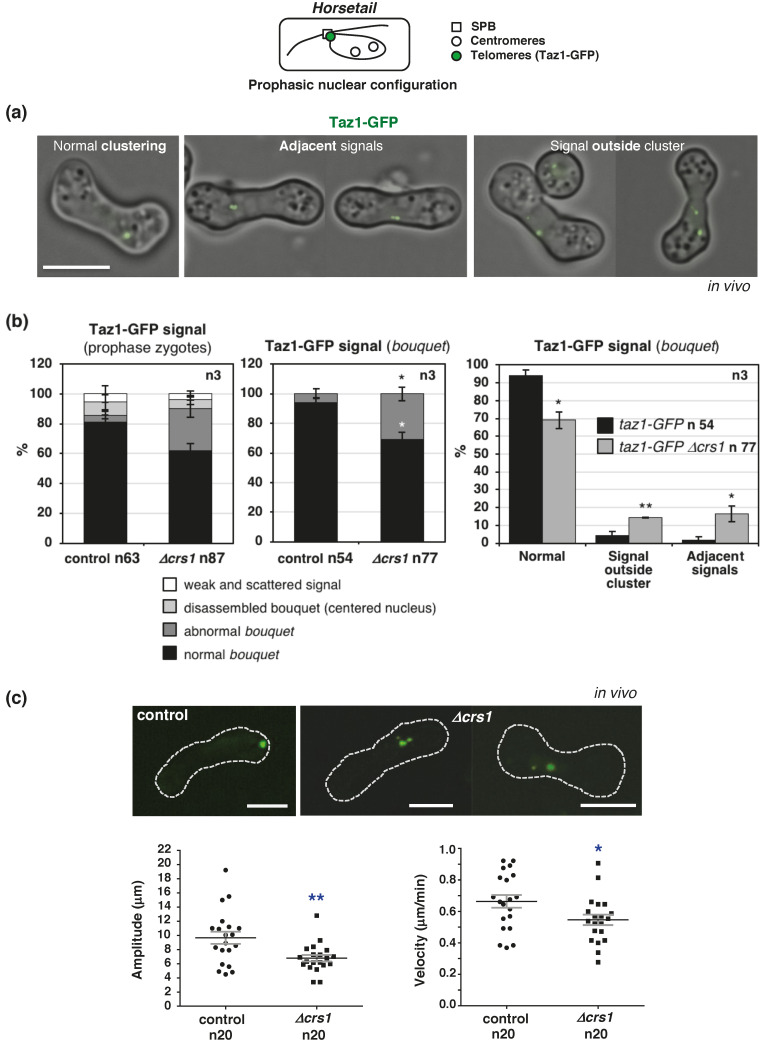
*Bouquet* formation and nuclear movement in *crs1* deletion mutants. Top panel: Schematic drawing of SPB, centromeres, and telomeres position during meiotic prophase is shown. (**a**) In vivo Taz1-GFP (telomeres) localization in prophase in *h^90^* wild-type (CMC1276) and *crs1* mutant (CMC1274) zygotes. Single plane bright-field images are overlapped to single plane Taz1-GFP images. Examples of zygotes with normal *bouquet* organization (normal Taz1-GFP clustering) and abnormal *bouquet* organization (adjacent Taz1-GFP signals and Taz1-GFP signals outside of the main cluster) are shown. (**b**) Left graph: Quantification of Taz1-GFP organization in wild-type and *crs1* deletion mutants. Data are the mean of the percentage of each category +/−SEM of three independent experiments with a total of 63 control and 87 *crs1* zygotes analyzed. In addition to “normal *bouquet*” and “abnormal *bouquet*” categories described in (**a**), similar low percentages of zygotes were observed in wild-type and *crs1* mutants with disassembled *bouquets* (centered rounded-nuclei with intense scattered Taz1-GFP signal), and very weak and scattered signal in more elongated nuclei. Middle graph: Quantification of *bouquet* organization (normal and abnormal categories) in the same experiments. Fifty-four control and 77 *crs1* zygotes analyzed. Right graph: Quantification of *bouquet* defects in wild-type and *crs1* deletion mutants in the same experiments. (**c**) Top images: Examples of cluster disorganization in *crs1* mutants (frames from time lapse experiments shown in Figures S3–S5). Bottom graphs: Quantification of the amplitude and velocity of nuclear movements in wild-type and *crs1* deletion mutants by Taz1-GFP tracking. Individual data are plotted, and the mean +/−SEM is indicated. Pooled data of two independent experiments with a total of 20 control and 20 *crs1* zygotes analyzed. *p* value * < 0.05, ** ≤ 0.01 (Student’s *t*-test, unpaired, two tails). Scale bar corresponds to 5 µm in all the images in the figure.

**Figure 8:**
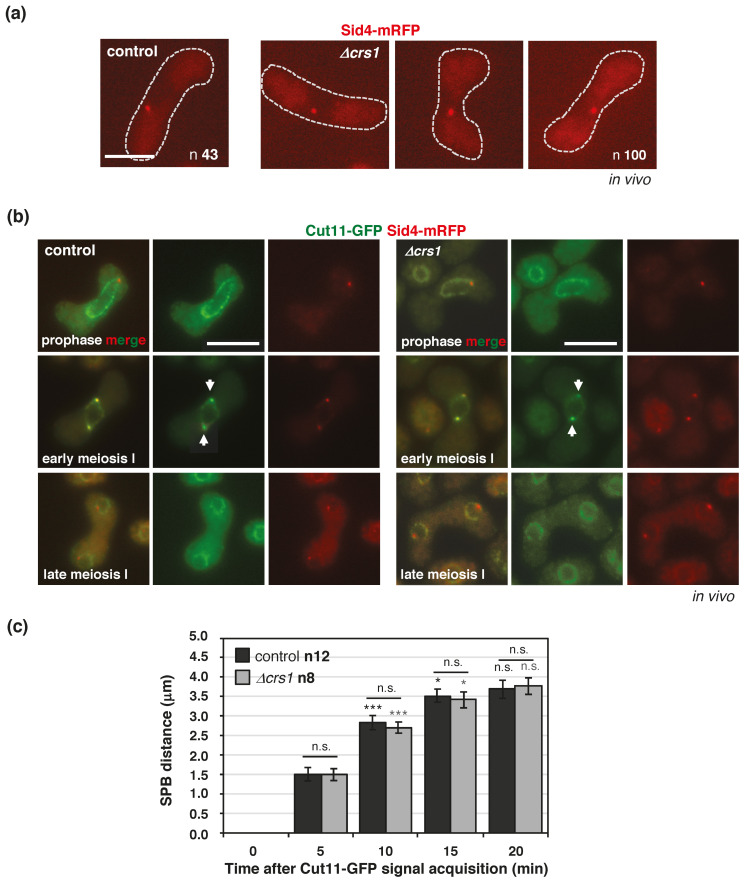
SPB insertion and separation in *crs1* deletion mutants. (**a**) In vivo Sid4-mRFP signal (SPB) in prophase in *h^90^* wild-type (CMC1027) and *crs1* mutant (CMC1388) zygotes. Pooled data of two independent experiments with a total of 43 control and 100 *crs1* zygotes analyzed. Images are single planes. (**b**) In vivo Cut11-GFP and Sid4-mRFP localization in prophase and meiosis I in *h^90^* wild-type (CMC1441) and *crs1* mutant (CMC1444) zygotes. Notice Cut11-GFP signal acquisition at the SPB (Sid4-mRFP) upon insertion in the nuclear membrane in early meiosis I (arrowheads). Images are either single planes or maximal projections of Z sections. (**c**) Quantification of SPBs separation after nuclear membrane insertion (Cut11-GFP signal acquisition) in time lapse experiments. Time 0 corresponds to the first time point in which Cut11-GFP was observed at the SPB (insertion). Pooled data of two independent experiments with a total of 12 control and 8 *crs1* zygotes analyzed. For each genotype, *p* value was calculated at each time point by comparison with the previous time point. In addition, control and *crs1* mutant were compared at each time point. No statistical differences between control and *crs1* mutant zygotes were observed. *p* value * < 0.05, *** ≤ 0.001 (Student’s *t*-test, unpaired, two tails). n. s. No statistically significant difference. Scale bar corresponds to 5 µm. Related to Videos S1–S4.

The localization of Crs1 at the SPB prompted us to check *bouquet* formation in *crs1* mutants using the telomeric Taz1-GFP protein [60]. Normal telomere-clustering was observed in most of *h^90^ taz1-GFP* control zygotes, 94% (n 54), meanwhile, in the *crs1* mutant the percentage was lower, 69% (n 77) (*p* value 0.0119) (Figure 7a,b). Thirty-one percent of zygotes showed either a Taz1-GFP signal outside of the main cluster (14.4%, *p* value 0.0099) or a less compacted cluster with adjacent Taz1-GFP foci (16.5%, *p* value 0.0361). These types of signals were observed in only 6% of the control cells. This phenotype was further analyzed by time lapse microscopy (Figure 7c), and examples of cluster instability during *horsetail* movement are shown in Figures S3–S5. We noticed that the movement of the cluster was impaired in those zygotes with detached telomeres, and also in zygotes without apparent cluster disorganization; therefore, we decided to analyze the nuclear movements in *crs1* mutants by tracking the motion of Taz1-GFP. Analysis of the amplitude of the movement of the cluster of telomeres, as well as velocity, showed that in *crs1* zygotes, the cluster moved less (amplitude 6.78 µm +/−0.46 SEM compared to 9.66 µm +/−0.86 SEM in the control, n 20, *p* value 0.0053) and at a slower speed (0.55 µm/min +/−0.033 SEM compared to 0.66 µm/min +/−0.041 SEM in the control, n 20, *p* value 0.0312) (Figure 7c). Only 5% of *crs1* zygotes showed a larger amplitude, and 10% a faster velocity than the control means, compared to 50% of the control zygotes. Thus, Crs1 seems to play a role in *bouquet* stability and nuclear movement. However, the integrity of the SPB, visualized with a Sid4-mRFP version, appeared normal in the mutant, indicating that the defects are not related to structural problems in the SPB (Figure 8a). Furthermore, we followed the SPB dynamics using a Cut11-GFP version, a nuclear envelope protein required for SPB insertion [61,62], and observed normal insertion (Cut11-GFP signal acquisition) and duplication of the SPB in the nuclear membrane in *crs1* mutant cells; moreover, SPBs separated with a kinetics similar to that of the control zygotes (Figure 8b,c and Supplementary Materials Videos S1–S4).

Given the location and role of Crs1 in telomere clustering, we decided to address whether Crs1 requires the *bouquet* configuration for proper localization. It has been shown that the localization of Cdc13 at the SPB in late prophase requires the SPB–telomere interaction [34], and we wonder if this would also be the case for Crs1. As shown in Figure S6, Crs1 appears in the SPB in *bqt1* mutants, with similar percentage of Sid4-mRFP colocalization to that observed in control zygotes, 74% (n 87) and 71% (n 95), respectively. Therefore, Crs1 does not require the SPB-telomere contact to localize at the SPB.

## 3. Discussion

Cyclins and CDKs are fundamental players in the biology of eukaryotic cells, with a central role in the control of mitotic and meiotic progression. The diversification of CDK complexes has specially flourished in higher eukaryotes, even though several complexes are also present in lower eukaryotes such as yeasts [63,64,65,66,67]. In fission yeast, it has been shown that a single Cyclin-CDK complex is sufficient to organize a “minimal” mitotic and meiotic cycle [3,4], suggesting that the additional complexes, and functions, originated from an ancestral component. The plasticity of CDK-complex adaptation is well exemplified in meiosis, where CDK complexes, already present in vegetative cells, have acquired meiotic functions in both lower and higher eukaryotes; and meiosis-specific variants have also emerged in both lower and higher eukaryotes (see Introduction for some examples).

In fission yeast, Crs1 was described as a meiosis-specific Cyclin-like protein Regulated via Splicing [27]. Among fission yeast cell cycle-cyclins, Crs1 shares with Puc1 the presence of a single cyclin-box domain at the amino-terminal part of the protein (Pombase, Protein features; [49]), instead of the common two-copy domain signature (amino-terminal box and carboxy-terminal box). This type of unconventional cyclin is common, and they are expressed in meiosis in other organisms [23,24]. However, there was no information about expression of the protein, associated kinase activity, or Cdc2 dependency. In this report we have shown that Crs1 exhibits kinase activity that depends on Cdc2, since the activity is strongly reduced in the *cdc2-33* mutant at the restrictive temperature (Figure 2). In addition, our results suggest a strong post-translational regulation since the kinase activity shows a biphasic pattern during meiotic progression, with a peak of activity early in S-phase and a second peak before meiosis I entry, meanwhile the protein is constantly present during this period of time (Figure 1 and Figure 3). This biphasic pattern of the kinase activity in meiosis is also shown for the Cig2 cyclin; however, in this case, the biphasic activity corresponds well with the RNA and the protein levels [32]. Given that during meiotic prophase Cdc2 is subject to the inhibitory phosphorylation of Tyr 15 [68], it is possible that Crs1-CDK complexes are under this regulation. In budding yeast meiosis, post-translational regulation of CDK activity has also been reported for cyclin Clb1 and Clb4-complexes [67,69,70].

Crs1 plays an important role during meiotic prophase, in the meiosis-specific event of programmed DSB formation, and therefore, in meiotic recombination [16]. The observation of a pan-nuclear localization of the protein—and Cdc2 dependent kinase activity—during prophase is compatible with this role of Crs1 (Figure 2, Figure 5 and Figure 6). However, in this phase of meiosis, Crs1 shows the lowest associated-kinase activity (Figure 3). Regarding DSB formation, CDK activity is absolutely necessary, and the predominant role of Crs1 in recombination cannot be fulfilled by other cyclins [16]. A CDK complex with low activity would be more feasibly regulated in an extremely flexible and responsive process such as DSB formation [71]; and, in addition, high levels of Crs1-CDK activity might be detrimental if an excess of DSBs are produced. On the other hand, it is formally possible that during meiotic prophase Crs1, somehow, loses its interaction with Cdc2, and the unbound protein performs its function in DSB formation.

In addition to the role in recombination, and in agreement with the first wave of associated kinase activity, Crs1 contributes to premeiotic DNA replication when the main G1/S cyclin Cig2 is absent, as indicated by the extremely slow progression through S-phase of *crs1 cig2* mutant cells (Figure 4 and Figure S2). This defect is stronger than the reported one for the triple *cig1 cig2 puc1* deletion mutant [4], indicating the important contribution of meiosis-specific cyclins in the process. Indeed, the meiosis-specific cyclin Rem1 also contributes to S-phase progression in the absence of Cig2; however, in this case, due to an advanced expression of *rem1* that is normally expressed temporarily later on in meiosis I entry [28]. Rem1 contribution seems even more important, since in the double *rem1 cig2* mutant, meiotic DNA replication is completely abolished.

Apart from the pan-nuclear localization of Crs1 during meiotic prophase, Crs1 is also present at the SPB (Figure 5, Figure 6 and Figure S6), and in *crs1* mutants, telomere-clustering at the SPB (*bouquet* organization) is less stable (Figure 7 and Figures S3–S5). However, SPB integrity, insertion in the nuclear membrane, and separation appear normal in *crs1* mutants (Figure 8, and Videos S1–S4). This phenotype, although less severe, is reminiscent of the defects observed in *bqt1* and *bqt2* mutants, defective in *bouquet* formation [37,38]. As for *bqt1* and *bqt2*, *crs1* gene expression is induced by pheromone signaling [37], the protein localizes at the SPB in early prophase, and it remains at SPBs in meiosis I [37,38] (Figure 5 and Figure 6). Thus, Crs1 might control telomere-clustering stability through the regulation of Bqt1 and Bqt2 proteins. *Bouquet* formation is required for chromosome alignment, promoting in this way recombination with the homologous chromosome and reducing ectopic recombination [41,42]. Therefore, this role of Crs1 could contribute, in addition to the control of DSB formation, to the recombination defect observed in *crs1* mutants [16]. It has been reported that 20% of *crs1 rem1* zygotes shows SPB fragmentation during abnormal chromosome segregations in meiosis I and/or meiosis II [4]. This defect is compatible with the localization of Crs1 at the SPB in meiosis I; however, we have not observed SPB fragmentation in *crs1* single mutants in our analysis (Figure 8 and Videos S1–S4), suggesting that the described defect is consequence of *rem1* deletion, which is normally expressed in meiosis I entry [28], or observed only in the double mutant. Murine CDK2, which is dispensable for mitotic cell divisions [72], is also required for telomere attachment to the nuclear envelope during meiotic prophase [17,73]. Interestingly, this role of CDK2 is shared by a conventional CDK complex (Cyclin E-CDK2), and by a non-conventional complex (Speedy A-CDK2) in which a meiosis-induced long CDK2-isoform associates with a non-cyclin atypical activator [18,19,31,74]. In this case, CDK2 locates at the attachment plates of the synaptonemal complex associated with the inner nuclear membrane [17].

Crs1 localization at the SPB is maintained in *bqt1* mutants (Figure S6), indicating that telomere contact is not required as reported for the localization of Cdc13 [34]. It will be interesting to explore Crs1-dependency of Bqt1/Bqt2 proteins for localization, as well as possible Crs1-CDK substrates among Bqt proteins and telomeric proteins. The telomeric protein Rap1, that bridges Taz1 with Bqt proteins at the SPB, is highly phosphorylated during meiotic prophase; and phosphorylation includes five CDK sites, four of them also phosphorylated in mitosis [75,76]. Phosphorylation in mitosis regulates the release of telomeres from the nuclear envelope prior to chromosome segregation; and phospho-mimic mutants show efficient sporulation, suggesting that *bouquet* formation is probably normal. Therefore, CDK phosphorylation of Rap1 seems compatible with normal *bouquet* organization; however, this phosphorylation does not regulate the interaction of Rap1 with Bqt1 and Bqt2 proteins [76].

In addition to the defect in telomere clustering stability, *crs1* mutants show a defective movement of the cluster (Figure 7c). Disorganization of the cluster of telomeres impinges on the movement of the nucleus since the attachment of chromosomes to the SPB is necessary to their motion [39]. However, *bqt* mutants impair the movement of the bulk of the nucleus, but not of the SPB that continues to oscillate between the cell poles [37,38]. This observation raises the possibility of an additional role for Crs1 in the control of the motion of the SPB, and therefore of the nucleus. Crs1 might regulate cytoplasmic events such as the organization of the astral microtubules or the loading of the dynein motor. Nevertheless, since the movement of the SPB has not been quantified in *bqt* mutants, we cannot exclude that *bouquet* configuration may be required for efficient SPB movement, and the *crs1* phenotype fully explained by the clustering defect.

The localization of Cdc2 during the sexual program is pretty dynamic. During conjugation and early karyogamy, in addition to a pan-nuclear signal, it is enriched at the telomeres–SPB–centromeres cluster, following the centromeres when they detach from the SPB during nuclear fusion. Cdc2 remains at centromeres during prophase and appears in the spindle in meiosis I [33]. In addition to the pan-nuclear and the centromere localization of Cdc2 during prophase, a weak signal it is also visible at the leading edge of the *horsetail* movement, as well as at the spindle poles in meiosis I, suggesting a SPB localization (see Figures 8 and 9 in [33]). This localization, that has been more recently confirmed [34], is compatible with the localization of Crs1, and supports the role of the Crs1-CDK complex in different meiotic processes. In addition, it is possible that other cyclins are also present in the SPB during meiotic prophase and meiosis I since at least Cdc13 and Cig2 accumulate at the SPB in vegetative cells [33], and in meiosis, Cdc13 is also highly enriched at the SPB from late prophase [34]. Nevertheless, Crs1 is the first cyclin for which the location in early meiosis is described. In addition, Crs1 is preserved at the SPB at late prophase and meiosis I (Figure 5b and Figure 6). Although we have not observed defects in SPB insertion in the mutant, Crs1 may contribute along with other cyclins to the proposed CDK function in nuclear envelope fenestration under the SPB, and therefore, to the assembly of the spindle [77,78]. Alternatively, Crs1 and the late recruitment of Cdc13 to the SPB might represent local CDK activation prior to meiosis I entry, like local CDK activity at the SPB in late G2 orchestrates mitosis entry in vegetative cells [79].

In this report, we have described that Crs1 (uncharacterized cyclin) shows Cdc2-associated kinase activity with a biphasic pattern during meiosis, unlike the protein, which is constantly present from S-phase to meiosis I. Apart from its previously described role in DSB formation, Crs1 also contributes to S-phase progression, and both functions are compatible with the pan-nuclear localization of the protein. In addition, Crs1 localizes at the SPB, where it stabilizes the *bouquet* configuration of the meiotic chromosomes and promotes proper SPB motion. Thus, the meiosis-specific Crs1 cyclin is a key regulatory factor involved in several meiotic events.

## 4. Materials and Methods

### 4.1. Yeast Manipulation and General Methods

Strains used are listed in Table S1, and they were obtained by meiotic crosses. Genetic crosses were done in Malt Extract plates with supplements (MEA-4S) at 25 °C. Cells were grown in Yeast Extract with Supplements (YES) or Edinburgh Minimal Medium (MM) with supplements at 32 °C or 25 °C. Supplements used in MM were Adenine and Leucine (225 mg/L) (Sigma, Saint Louis, MO, USA). YES supplemented with 0.1 mg/mL G-418 or Hygromycin B (ForMedium, Norfolk, UK) was used to select and follow deletion mutants, and GFP, Cherry, and mRFP-tagged gene versions. Recombination assays were done in MEA-4S at 25 °C as described in [16]. Diploid *pat1-114* (or *pat1-as1*) *leu1-32* strains were obtained by protoplast fusion and selection for complementation of *ade6-M210* and *ade6-M216* alleles [80]. Synchronous meiosis by thermal inactivation at 34 °C of the *pat1-114* temperature-sensitive allele and cell collection for flow cytometry analysis were done as previously described [81]. In the case of *pat1-as1 cdc2-33* meiosis, synchrony was induced by Pat1-as1 inactivation adding the 3-MB-PP1 ATP-analog (Toronto Research Chemicals Inc., Toronto, ON, Canada) at the beginning of the kinetics (minus Nitrogen depleted cells) to 25 µM final concentration. Becton Dickinson FACSCalibur and CellQuest software (Becton Dickinson, Franklin Lakes, NJ, USA) were used for cell acquisition and data analysis; 10^4^ events were scored at each time point. Chromosome segregations were followed by DAPI staining of ethanol fixed cells and counting the number of nuclei; 300 cells were scored at each time point. For Western blot, 1.5 × 10^8^ cells were collected at different time points during the meiotic time-course, and protein extracts were prepared in trichloroacetic acid (TCA) [82]. Proteins were detected with primary anti-GFP (monoclonal JL-8, Living colors, Clontech Laboratories Inc., Mountain View, CA, USA) and anti-Actin (monoclonal clone C4, MP Biomedicals LLC, Solon, OH, USA), and secondary anti-mouse light chain-specific horseradish peroxidase-conjugated (115-035-174 Jackson ImmunoResearch Laboratories Inc., West Grove, PA, USA) antibodies. Crs1-GFP signal was developed with SuperSignal West DURA extended Kit (Pierce, Rockford, IL, USA), and Actin signal with ECL Western Blotting Kit (Amersham, GE Healthcare UK, Buckinghamshire, UK).

### 4.2. Crs1-GFP Fusion

Oligos *crs1-SalI* (ACTGGTCGACGTAATGAAGGG) and *crs1-BamHI* (CCGGGGATCCGTGCTAACATATCCG) were used to amplify from genomic DNA a fragment of 283 bp just upstream of the STOP codon, and oligos *crs1-EcoRI* (GCTCGAATTCAGCTTCAAACCC) and *crs1-EcoRV* (TTCAGATATCCGCTAGCTGCTG) were used to amplify a fragment of 281 bp just downstream of the STOP codon. PCR fragments were cloned into plasmid *pFA6a-GFP-KanMX6* to generate plasmid *pFA6a-up crs1-GFP-KanMX6-down crs1* (CMC41). Cloned fragments were checked by sequencing, and the cassette for transformation was obtained by PCR with oligos *crs1-HAC1* (TATCGAATATAGGCCGACGG) and *crs1-C2* (GTTTACCGACTGCGCCTGCC). Strain *h^90^ 968* (CMC3) was transformed to G418 resistance using the Lithium Acetate protocol [83]. Correct integration was checked by PCR and sequencing, and the required strains were obtained by meiotic crosses.

### 4.3. Kinase Assays

At the required meiotic time-points 3 × 10^8^ cells were collected at 4 °C, washed with 1 mL cold STOP Buffer (0.9% NaCl, 1 mM NaN_3_, 10 mM EDTA, 50 mM NaF), and frozen until processing. Protein extracts were prepared using HB Buffer (25 mM MOPS, 60 mM β-Glycerophosphate, 15 mM MgCl_2_, 1 mM DTT, 5 mM p-Nitrophenyl Phosphate, 15 mM EGTA, 1% Triton X-100) supplemented with 2X protease inhibitors (Complete Protease Inhibitor Cocktail, Roche Diagnostic GmbH, Mannheim, Germany), 1 mM PMSF, and 1 mM Sodium Orthovanadate. Samples of 2 mg (BCA quantification) were immunoprecipitated at 0 °C for 1.5 h using 1 µL of anti-GFP polyclonal antibodies (A6455 Invitrogen, Paisley, UK), 2 µL of SP4 anti-Cdc13 polyclonal antibodies [51], or 2 µL of Cg14 anti-Cig2 polyclonal antibodies [84], and kinase assays were done as previously described [84]. To test Cdc2-dependent activity, immunoprecipitates were split in two in the last wash just before being assayed in parallel at 25 °C and 38 °C. Activity quantification was done with Quantity One software (Bio-Rad, Hercules, CA, USA) and under-saturated phosphorimager exposures (PMI Personal Molecular Images, Bio-Rad; Fuji imaging BAS-III screens, Fuji Film, Tokyo, Japan). The signal of an equal size window below the corresponding H1 band was subtracted for background correction of the individual bands. In the kinetics, the corrected signal in the minus nitrogen time point was subtracted from the rest of the corrected bands, and the activity related to the time point of maximal activity.

### 4.4. Microscopy

For visualization of live *h^90^* zygotes, cells were grown at 25 °C first in YES liquid until exponential phase, next diluted to MM and grown until exponential phase, and then collected, washed 3 times with sterile water, and transferred to MM without nitrogen at 1.5 O. D. for an overnight incubation (14 h) with low agitation. Next day, the culture was sonicated to disrupt cell aggregates, and cells placed in a poly-L-lysine (Sigma) coated slide for microscope observation. For time lapse experiments, the cultures were diluted 1/7 in the same medium and 300 µL placed in a chamber (Slide 8 well for Live Cell Analysis, Ibidi GmbH, Martinsried, Germany) coated with 10 µL of 2 mg/mL soybean Lectin (Sigma). Cells were allowed to stick to the bottom of the chamber for 5 min before removing the medium, and washed twice with new medium. For experiments in Figure 5, Figure 6 and Figure S6, and Videos S1–S4, cells were observed under an Olympus IX81 spinning disk microscope (Olympus, Tokyo, Japan), equipped with a confocal CSUX1-A1 module (Yokogawa, Tokyo, Japan), a 100X/1.4 Oil Plan APO lens, an Evolve camera (Teledyne Photometrics, Tucson, AZ, USA), and Metamorph software (Molecular Devices LLC, San Jose, CA, USA). Crs1-GFP signal was collected with 2000–2500 ms of exposure time at 100% laser power, and Sid4-mRFP [57] and Cnp1-Cherry [58] signal were collected with 200 ms of exposure time at 50% laser power. In time lapse experiments with Sid4-mRFP and Cut11-GFP [85], signals were collected with 500 ms exposure time at 75% and 50% laser power, respectively. For experiments in Figure 7a,b and Figure 8a,b, signals were observed under a Nikon Eclipse 90i microscope equipped with a 100X/1.4 Oil Plan APO VC lens (Nikon, Tokyo, Japan), a Hamamatsu ORCA-ER camera (Hamamatsu Photonics KK, Hamamatsu City, Japan, and Metamorph software (Molecular Devices LLC). Taz1-GFP [60] signal was collected with 100 ms exposure time at 50% led power, Sid4-mRFP with 400 ms at 100% led power, and Cut11-GFP with 200 ms at 50% led power. For time lapse experiments in Figure 7c, and Figures S3–S5, cells were observed under a Nikon Ti2-E spinning disk equipped with a confocal Dragonfly module (ANDOR, Belfast, Northern Ireland), a 100X/1.45 Oil Plan APO lens, a sCMOS Sona 4.2B-11 camera (ANDOR), and Fusion 2.2 software (ANDOR). Taz1-GFP signal were collected with 200 ms of exposure time at 50% power laser. In all cases, 9–11 Z sections were taken at 0.5–0.4 µm step size to cover 4 µm of total thickness, and for time lapse experiments the microscope chambers were kept at 25 °C and images taken every 5 min. Images were analyzed using MetaMorph (Molecular Devices LLC) or Fiji [86] software. Amplitude of the movement and velocity of the cluster of telomeres (Figure 7c) were calculated using time lapse experiments shown in Figures S3–S5, and the Manual Tracking plugin of Fiji. Control and *crs1* zygotes showed similar variation in Z for the Taz1-GFP signal and, therefore, maximal projections were used. In the case of zygotes with cluster disorganization, the main Taz1-GFP signal was used to follow the cluster. Zygotes with 16–25 time points (75–120 min time lapse) before cluster resolution were analyzed, with an average of 23 frames per zygote in both genotypes. Amplitude was calculated as the different between most distal positions at both ends of the zygote, using the mean of the four most distal positions at each end. Velocity was calculated as the mean of the velocities for each time point interval in the time lapse. Measurement of SPB separation in Figure 8 was done using the experiments shown in Videos S1–S4, maximal projections of the Cut11-GFP signal, and Fiji software. Duplicated SPBs are in close focal planes (+/−1 Z), and the distance between the two SPBs steadily increased with time in the control, indicating the reliability of the method.

## Data Availability

Not applicable.

## Supplementary Materials

The following are available online at https://www.mdpi.com/article/10.3390/ijms22115483/s1, Figure S1: *Cig2* but not Cdc13 shows thermosensitive kinase activity, Figure S2: Meiosis progression of *cig2* and *crs1* mutants, Figure S3: Time lapse of *h^90^ taz1-GFP* zygote, Figure S4: Time lapse of *h^90^ taz1-GFP crs1* zygote, Figure S5: Time lapse of *h^90^ taz1-GFP crs1* zygote, Figure S6: In vivo Crs1-GFP and Sid4-mRFP localization in *bqt1* mutants, Table S1: *S. pombe* strains, Video S1: Time lapse of *h^90^ sid4-mRFP cut11-GFP* zygotes, Video S2: Time lapse of *h^90^ sid4-mRFP cut11-GFP* zygotes, Video S3: Time lapse of *h^90^ sid4-mRFP cut11-GFP crs1* zygotes, Video S4: Time lapse of *h^90^ sid4-mRFP cut11-GFP crs1* zygotes.
