# Supplementary material for: The Meiosis-Specific Crs1 Cyclin Is Required for Efficient S-Phase Progression and Stable Nuclear Architecture
Source: Int J Mol Sci. 2021 May 22;22(11):5483. doi: 10.3390/ijms22115483 (PMC8196990; doi:10.3390/ijms22115483)
Supplement: Supplementary file 1 [file ijms-22-05483-s001.zip › Supplementary Figure legends.pdf]

Supplementary Figure legends

Figure S1: Cig2 but not Cdc13 shows thermosensitive kinase activity.
In vitro kinase assays (histone H1 substrate) of Cdc13 and Cig2 immunoprecipitates (IP) at meiotic prophase (5 hr after meiotic induction) of synchronous diploid *pat1-as1(L95G) crs1-GFP* control (CMC1098) and *cdc2-33* (CMC1109) meiosis at 25ºC; 3-MB-PP1 ATP-analog added at the beginning of meiotic induction (kinetics shown in Figure 2a). IPs were split in two just before the assay, and tested for kinase activity at 25ºC and 38ºC (permissive and restrictive temperature for *cdc2-33* mutation). **Top panel** shows phosphorimager scanning of the assays ran in an acrylamide gel, and percentages of activity. **Bottom panel** shows Coomassie staining of the same gel. Graphs **on the right** represent Cdc13 and Cig2-associated prophasic-kinase activity as the percentage of the activity observed in the *cdc2*$^{+}$ control at 25ºC. Related to Figure 2.

Figure S2: Meiosis progression of *cig2* and *crs1* mutants.
a) Flow cytometry analysis of synchronous diploid *pat1-114* meiosis of control (CMC1074), *crs1* (CMC1059), *cig2* (CMC1022), double *crs1 cig2* (CMC1131) deletion mutants, and two independent *crs1 cig2* clones. DNA content (FL2-H) histograms are shown. Dashed-lined box outlines S-phase progression. **(b)** Quantification of chromosome segregation by DAPI staining and nuclear counting (1 nucleus, 2 nuclei, 3 nuclei, and 4 nuclei) is shown. The arrows indicate meiosis I (MI) entry, and the vertical dashed-lines indicate the peak of MI. Related to Figure 4.

Figure S3: Time lapse of *h*^{90} *taz1-GFP* zygote.
Frames of a time lapse experiment showing *bouquet* organization (Taz1-GFP telomere clustering) in a control zygote. Time (minutes) from the beginning of the experiment is indicated. Images are maximal projections of 9 Z sections (0.5 µm step size). Scale bar corresponds to 5 µm. **Bottom graph** Representation of the movement of the cluster. Related to Figure 7.

Figure S4: Time lapse of *h*^{90} *taz1-GFP crs1* zygote.
Frames of a time lapse experiment showing *bouquet* organization (Taz1-GFP telomere clustering) in a *crs1* mutant zygote. Time (minutes) from the beginning of the experiment is indicated. Images are maximal projections of 9 Z sections (0.5 µm step size). Scale bar corresponds to 5 µm. **Bottom graph** Representation of the movement of the cluster. Related to Figure 7.

Figure S5: Time lapse of *h*^{90} *taz1-GFP crs1* zygote.
Frames of a time lapse experiment showing *bouquet* organization (Taz1-GFP telomere clustering) in a *crs1* mutant zygote. Time (minutes) from the beginning of the experiment is indicated. Images are maximal projections of 9 Z sections (0.5 µm step size). Scale bar corresponds to 5 µm. **Bottom graph** Representation of the movement of the cluster. Related to Figure 7.

Figure S6: *In vivo* Crs1-GFP and Sid4-mRFP localization in *bqt1* mutants.
In vivo Crs1-GFP and Sid4-mRFP signal (SPB component) co-localization in prophase in *h*^{90} wild-type (CMC1076) and *bqt1* mutant (CMC1671) zygotes. Images are single planes. Bright-field images on the left help to localize the nucleus, which appears as a smooth area more or less stretched depending on the nuclear movement in a rugged context. 71% of control zygotes with a single Sid4-mRFP signal showed Crs1-GFP co-localization (n 95), and 74% of *bqt1* zygotes (n 87). Scale bar corresponds to 5 µm.
