# Supplementary material for: The Meiosis-Specific Crs1 Cyclin Is Required for Efficient S-Phase Progression and Stable Nuclear Architecture
Source: Int J Mol Sci. 2021 May 22;22(11):5483. doi: 10.3390/ijms22115483 (PMC8196990; doi:10.3390/ijms22115483)
Supplement: Supplementary file 1 [file ijms-22-05483-s001.zip › Table S1.pdf]

Strain	Genotype	Used in
**CMC3**	*h90 968*	Crs1-GFP construction
**CMC381**	h^{+} *ade6-3049*	Fig. 1a
**CMC987**	h^{-} *ade6-M26 crs1-GFP::KanMX6*	Fig. 1a
**CMC988**	h^{+} *ade6-3049 crs1-GFP::KanMX6*	Fig. 1a
**CMC1000**	h^{-}/h^{-} *pat1-114/pat1-114 ade6-M210/ade6-M216 leu1-32/leu1-32 crs1-GFP::KanMX6/crs1-GFP::KanMX6*	Fig. 1b-c, Fig. 3 and Fig. 5b
**CMC1001**	h^{-} *ade6-M26*	Fig. 1a
**CMC1022**	h^{-}/h^{-} *pat1-114/pat1-114 ade6-M210/ade6-M216 leu1-32/leu1-32 ura4-D18/ura4-D18 cig2::ura4*$^{+}$*/cig2::ura4*$^{+}$	Fig. 4 and Fig. S2
**CMC1027**	h^{90} *sid4-mRFP::Kan*	Fig. 8a
**CMC1059**	h^{-}/h^{-} *pat1-114/pat1-114 ade6-M210/ade6-M216 leu1-32/leu1-32 crs1::hphMX6/crs1::hphMX6*	Fig. 4 and Fig. S2
**CMC1073**	h^{90} *cnp1-cherry::Kan crs1-GFP::KanMX6*	Fig. 6
**CMC1074**	h^{-}/h^{-} *pat1-114/pat1-114 ade6-M210/ade6-M216 leu1-32/leu1-32*	Fig. 1b, Fig. 4, and Fig. S2.
**CMC1076**	h^{90} *sid4-mRFP::Kan crs1-GFP::KanMX6*	Fig. 5a
**CMC1098**	h^{-}/h^{-} *pat1-as1(L95G)::KanMX6/pat1-as1(L95G)::KanMX6 leu1-32/leu1-32 ade6-M210/ade6-M216 crs1-GFP::KanMX6/crs1-GFP::KanMX6*	Fig. 2 and Fig. S1
**CMC1109**	h^{-}/h^{-} *pat1-as1(L95G)::KanMX6/pat1-as1(L95G)::KanMX6 leu1-32/leu1-32 ade6-M210/ade6-M216 crs1-GFP::KanMX6/crs1-GFP::KanMX6 cdc2-33/cdc2-33*	Fig. 2 and Fig. S1
**CMC1131**	h^{-}/h^{-} *pat1-114/pat1-114 ade6-M210/ade6-M216 leu1-32/leu1-32 ura4-D18/ura4-D18 crs1::hphMX6/crs1::hphMX6 cig2::ura4*$^{+}$*/cig2::ura4*$^{+}$	Fig. 4 and Fig. S2
**CMC1274**	h^{90} *crs1::hphMX6 lys1*$^{+}$*::taz1-GFP taz1::ura4*$^{+}$ *ura4-D18*	Fig. 7, Fig. S4 and Fig. S5
**CMC1276**	h^{90} *lys1*$^{+}$*::taz1-GFP taz1::ura4*$^{+}$ *ura4-D18*	Fig. 7 and Fig. S3
**CMC1388**	h^{90} *sid4-mRFP::Kan crs1::hphMX6*	Fig. 8a
**CMC1441**	h^{90} *sid4-mRFP::Kan leu1-32::integrant pJK148 cut11-GFP*	Fig. 8b-c, Video 1 and Video 2
**CMC1444**	h^{90} *crs1::hphMX6 sid4-mRFP::Kan leu1-32::integrant pJK148 cut11-GFP*	Fig. 8b-c, Video 3 and Video 4
**CMC1671**	h^{90} *bqt1::KanMX6 sid4-mRFP::Kan crs1-GFP::KanMX6*	Fig. S6

Table S1. *S. pombe* strains
Alleles other than commonly used auxotrophies and mating type are *pat1-114* [48], *pat1-as1(95G)::KanMX6* [53], *cdc2-33* [52], *cig2::ura4*$^{+}$ [56], *crs1::hphMX6* [16], *sid4-mRFP::Kan* [57], *cnp1-cherry::Kan* [58], *leu1-32::integrant pJK148 cut11-GFP* [85], *crs1-GFP::KanMX6* was generated in this study (Materials and Methods).
