# Supplementary material for: The Meiosis-Specific Crs1 Cyclin Is Required for Efficient S-Phase Progression and Stable Nuclear Architecture
Source: Int J Mol Sci. 2021 May 22;22(11):5483. doi: 10.3390/ijms22115483 (PMC8196990; doi:10.3390/ijms22115483)
Supplement: Supplementary file 1 [file ijms-22-05483-s001.zip › Video legends final version.pdf]

Video legends

Video 1: Time lapse of *h^{90} sid4-mRFP cut11-GFP* zygotes.
Time lapse experiment showing SPB insertion in the nuclear membrane in control zygotes. Notice Cut11-GFP signal acquisition at the SPB (Sid4-mRFP) upon nuclear membrane insertion. Images were taken every 5 minutes; frames correspond to the overlay of the maximal projections for each channel (9 Z sections, 0.5 µm step size). Related to Figure 8.

Video 2: Time lapse of *h^{90} sid4-mRFP cut11-GFP* zygotes.
Time lapse experiment showing SPB insertion in the nuclear membrane in control zygotes. Notice Cut11-GFP signal acquisition at the SPB (Sid4-mRFP) upon nuclear membrane insertion. Images were taken every 5 minutes; frames correspond to the overlay of the maximal projections for each channel (9 Z sections, 0.5 µm step size). Related to Figure 8.

Video 3: Time lapse of *h^{90} sid4-mRFP cut11-GFP crs1* zygotes.
Time lapse experiment showing SPB insertion in the nuclear membrane in *crs1* mutant zygotes. Notice Cut11-GFP signal acquisition at the SPB (Sid4-mRFP) upon nuclear membrane insertion. Images were taken every 5 minutes; frames correspond to the overlay of the maximal projections for each channel (9 Z sections, 0.5 µm step size). Notice the segregation defect in the upper zygote (visualized as an extra nuclear mass outlined by the nuclear membrane marker Cut11-GFP). Related to Figure 8.

Video 4: Time lapse of *h^{90} sid4-mRFP cut11-GFP crs1* zygotes.
Time lapse experiment showing SPB insertion in the nuclear membrane in *crs1* mutant zygotes. Notice Cut11-GFP signal acquisition at the SPB (Sid4-mRFP) upon nuclear membrane insertion. Images were taken every 5 minutes; frames correspond to the overlay of the maximal projections for each channel (9 Z sections, 0.5 µm step size). Related to Figure 8.
