# Supplementary figures and images for: The Meiosis-Specific Crs1 Cyclin Is Required for Efficient S-Phase Progression and Stable Nuclear Architecture
Source: Int J Mol Sci. 2021 May 22;22(11):5483. doi: 10.3390/ijms22115483 (PMC8196990; doi:10.3390/ijms22115483)

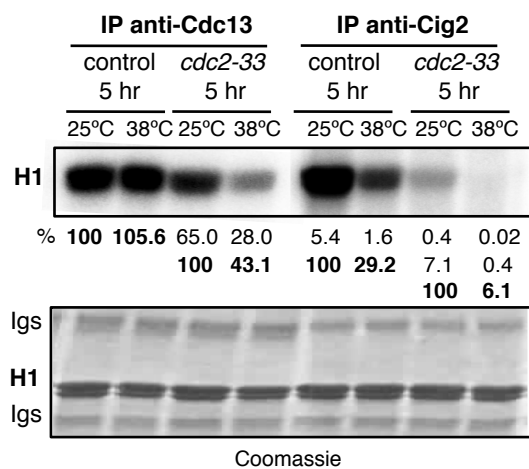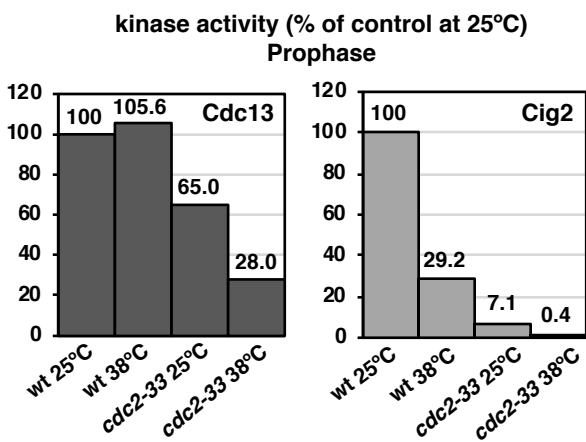

Supplement: Supplementary file 1 [file ijms-22-05483-s001.zip › Fig S1.pdf]

(a)

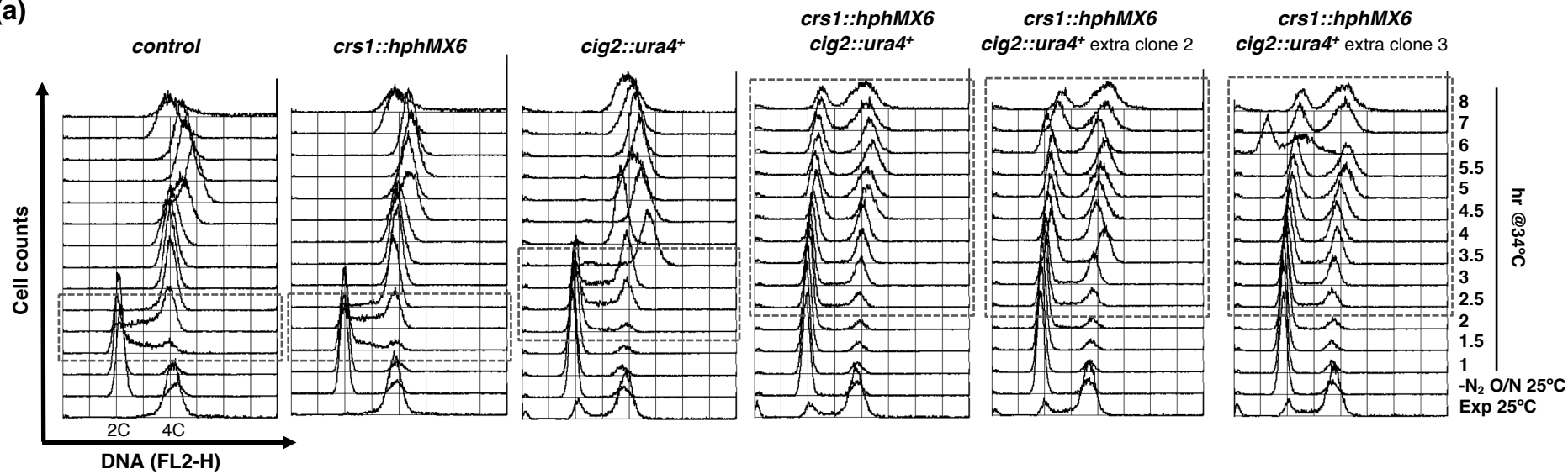

(b)

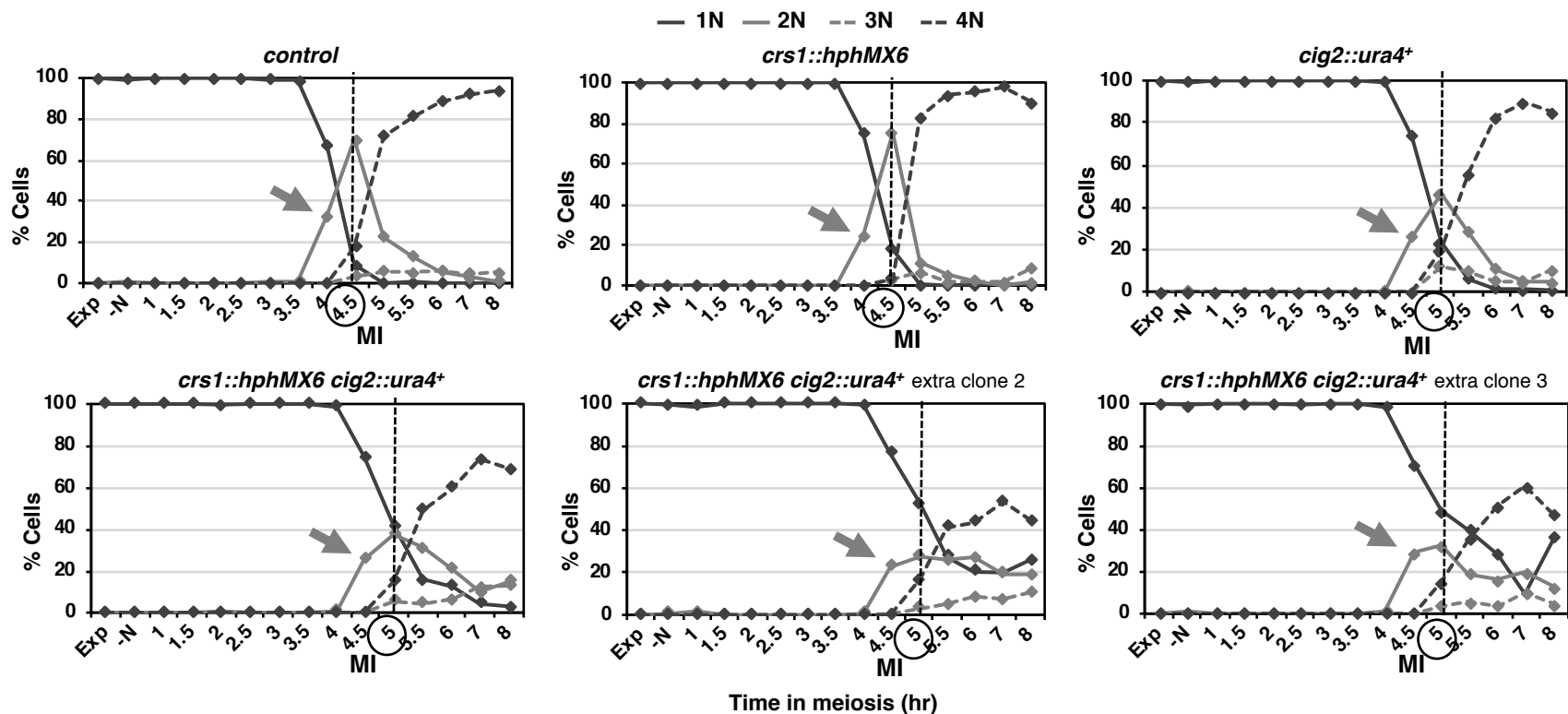

Supplement: Supplementary file 1 [file ijms-22-05483-s001.zip › Fig S2.pdf]

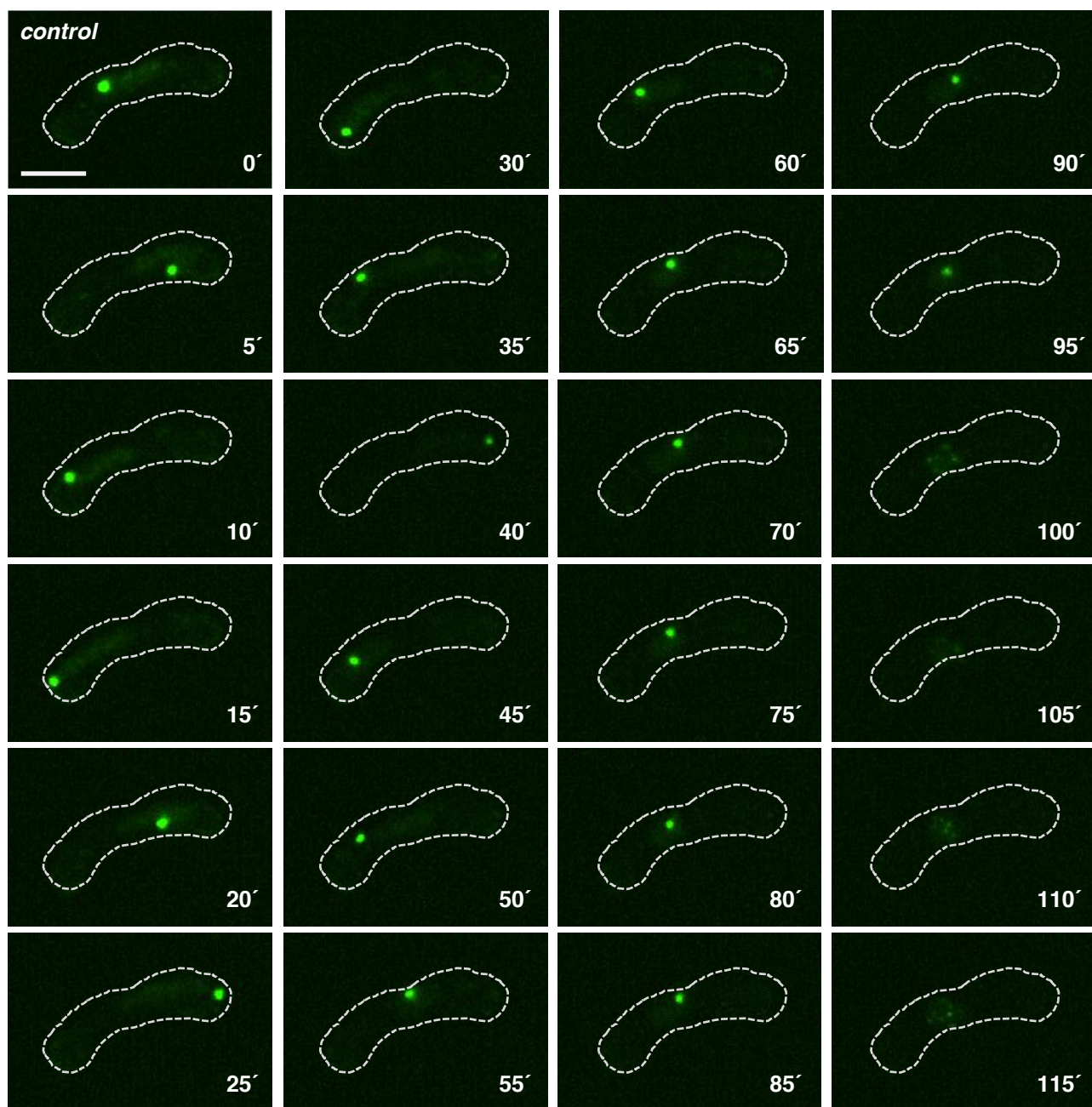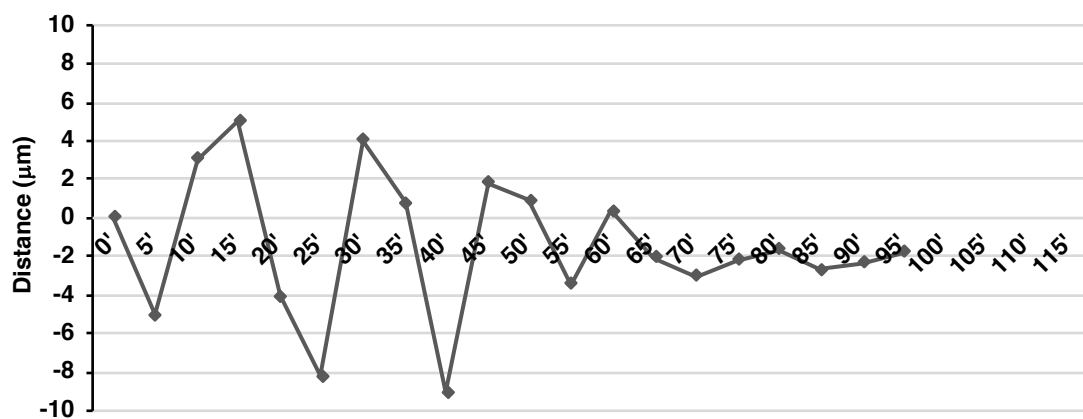

Supplement: Supplementary file 1 [file ijms-22-05483-s001.zip › Fig S3.pdf]

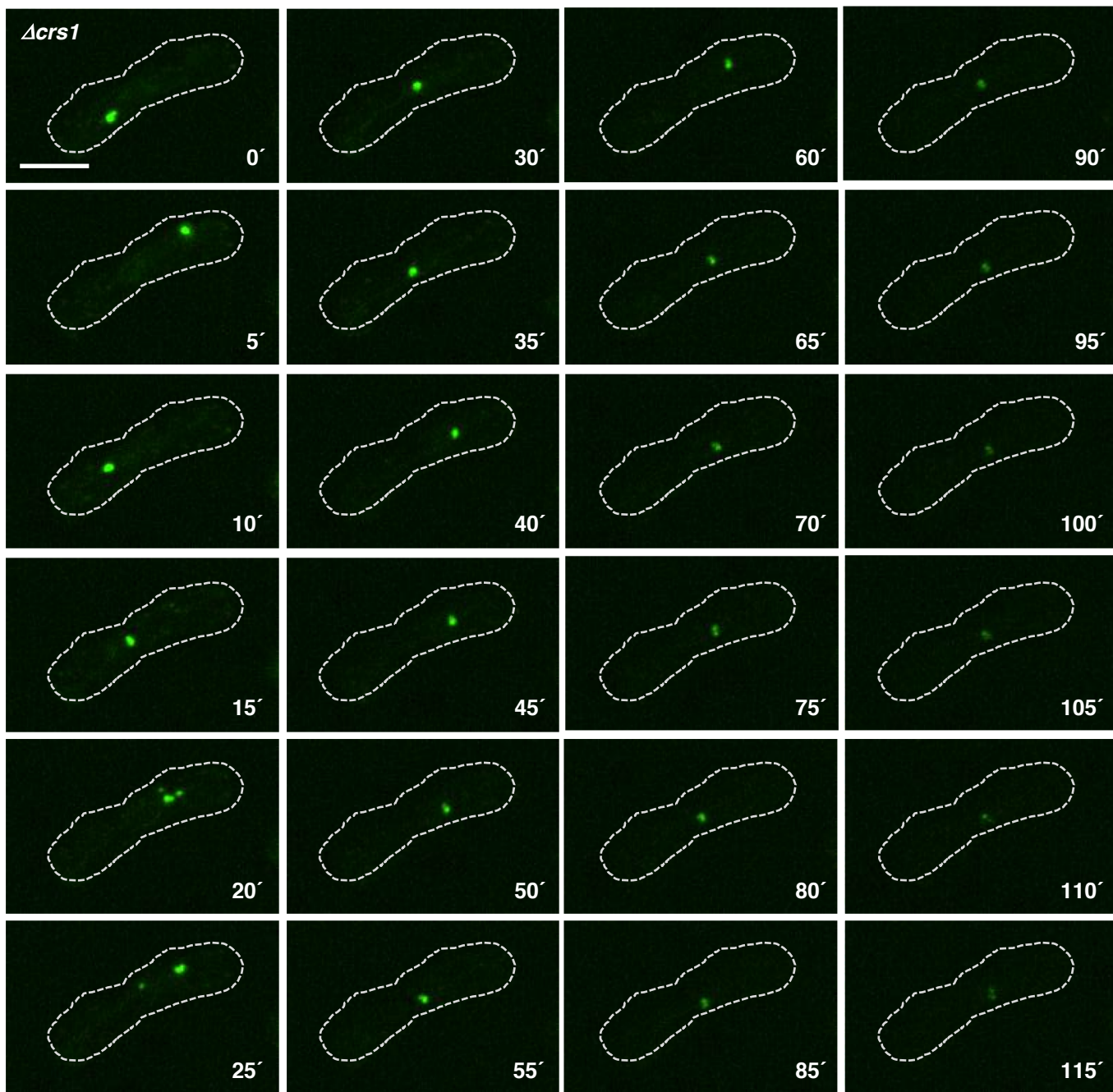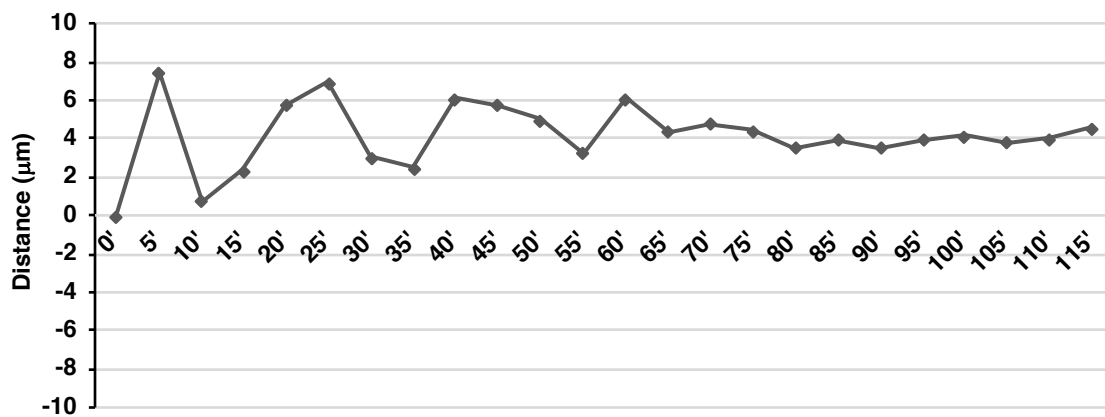

Supplement: Supplementary file 1 [file ijms-22-05483-s001.zip › Fig S4.pdf]

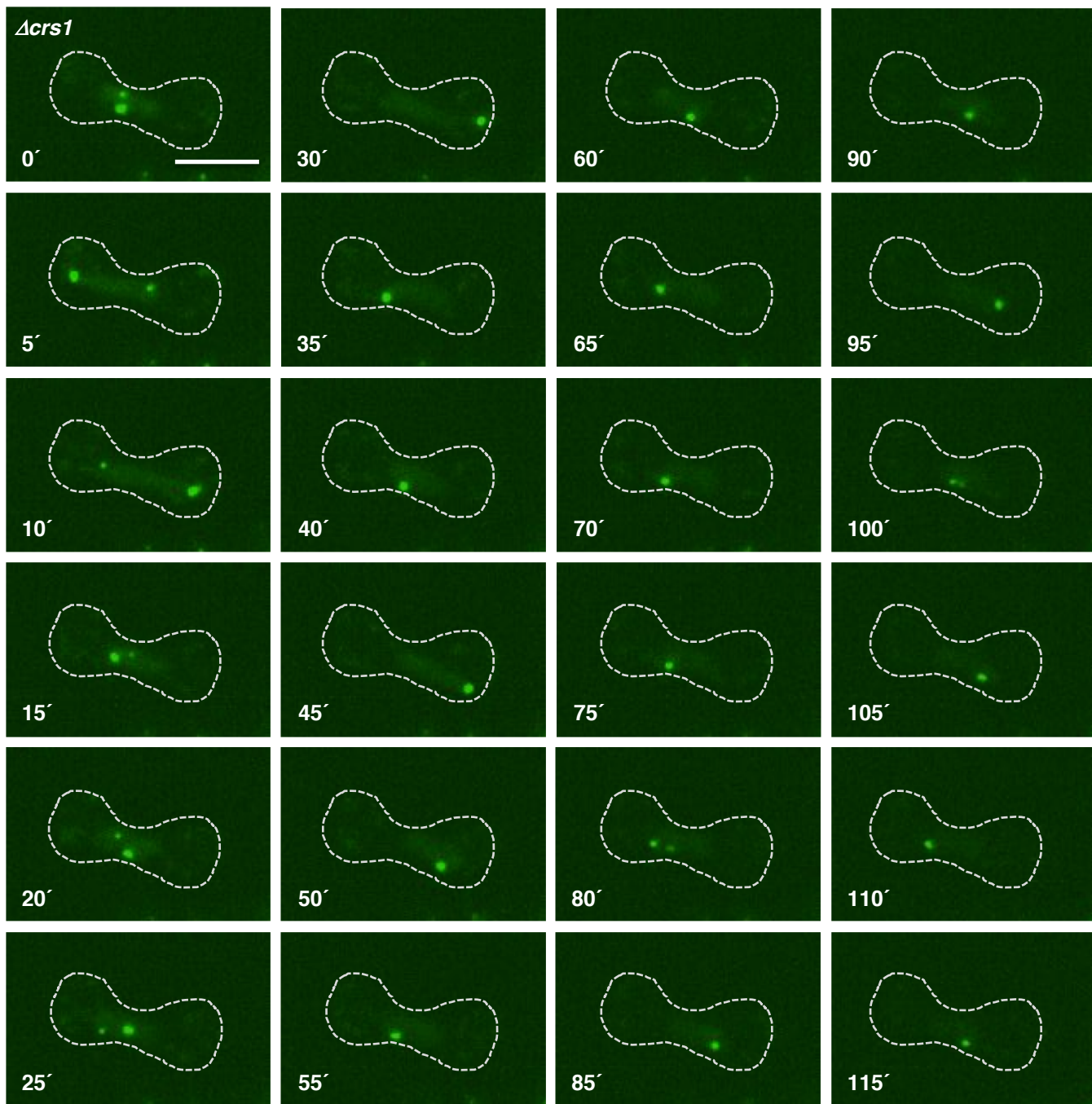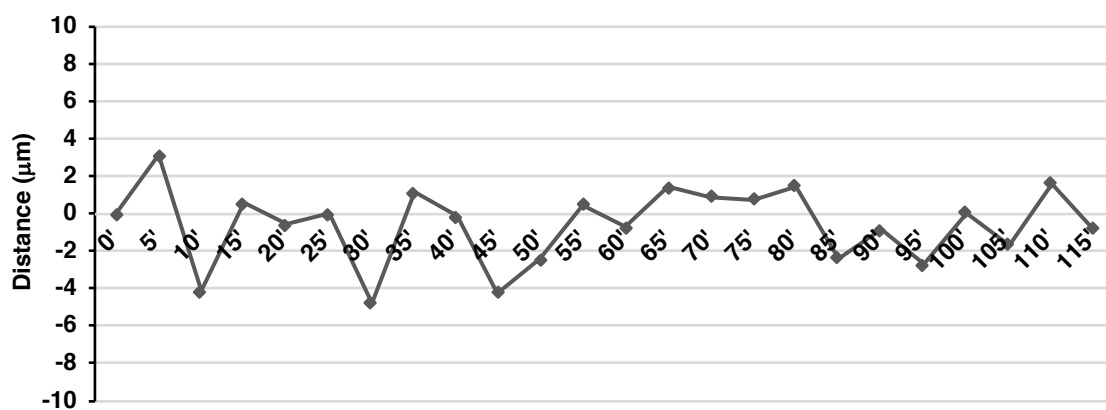

Supplement: Supplementary file 1 [file ijms-22-05483-s001.zip › Fig S5.pdf]

*in vivo*

Crs1-GFP Sid4-mRFP

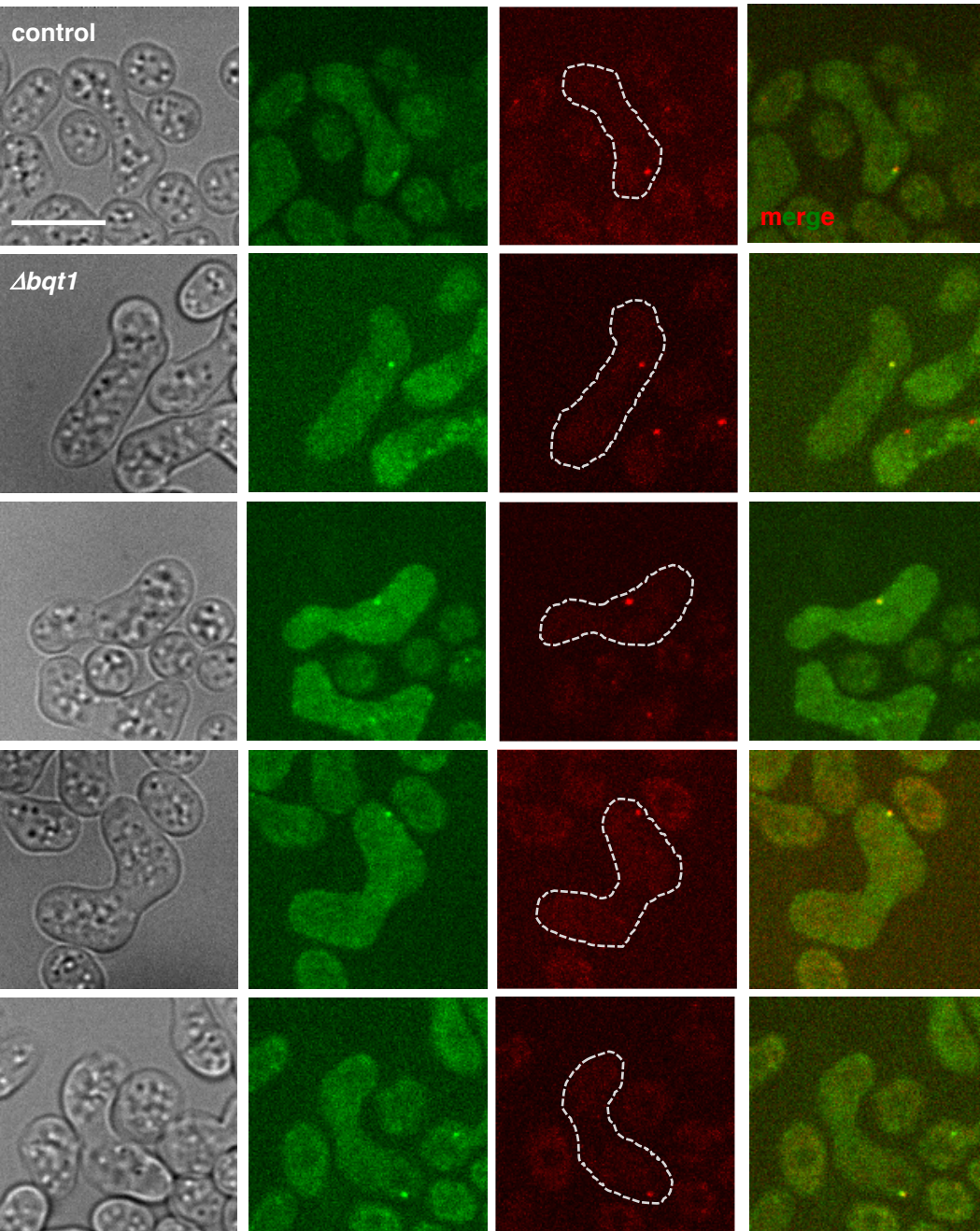

Supplement: Supplementary file 1 [file ijms-22-05483-s001.zip › Fig S6.pdf]
